# The Gastrointestinal Tract as a Key Target Organ for the Health-Promoting Effects of Dietary Proanthocyanidins

**DOI:** 10.3389/fnut.2016.00057

**Published:** 2017-01-03

**Authors:** María José Cires, Ximena Wong, Catalina Carrasco-Pozo, Martin Gotteland

**Affiliations:** ^1^Faculty of Medicine, Department of Nutrition, University of Chile, Santiago, Chile; ^2^Institute of Nutrition and Food Technology (INTA), University of Chile, Santiago, Chile

**Keywords:** proanthocyanidins, gastrointestinal tract, *Helicobacter pylori*, intestinal microbiota, digestive enzymes

## Abstract

Proanthocyanidins (PACs) are polymers of flavan-3-ols abundant in many vegetable foods and beverages widely consumed in the human diet. There is increasing evidence supporting the beneficial impact of dietary PACs in the prevention and nutritional management of non-communicable chronic diseases. It is considered that PACs with a degree of polymerization >3 remain unabsorbed in the gastrointestinal (GI) tract and accumulate in the colonic lumen. Accordingly, the GI tract may be considered as a key organ for the healthy-promoting effects of dietary PACs. PACs form non-specific complexes with salivary proteins in mouth, originating the sensation of astringency, and with dietary proteins, pancreatic enzymes, and nutrient transporters in the intestinal lumen, decreasing the digestion and absorption of carbohydrates, proteins, and lipids. They also exert antimicrobial activities, interfering with cariogenic or ulcerogenic pathogens in the mouth (*Streptococcus mutans*) and stomach (*Helicobacter pylori*), respectively. Through their antioxidant and antiinflammatory properties, PACs decrease inflammatory processes in animal model of gastric and colonic inflammation. Interestingly, they exert prebiotic activities, stimulating the growth of *Lactobacillus* spp. and *Bifidobacterium* spp. as well as some butyrate-producing bacteria in the colon. Finally, PACs are also metabolized by the gut microbiota, producing metabolites, mainly aromatic acids and valerolactones, which accumulate in the colon and/or are absorbed into the bloodstream. Accordingly, these compounds could display biological activities on the colonic epithelium or in extra-intestinal tissues and, therefore, contribute to part of the beneficial effects of dietary PACs.

## Introduction

**Figure 1:**
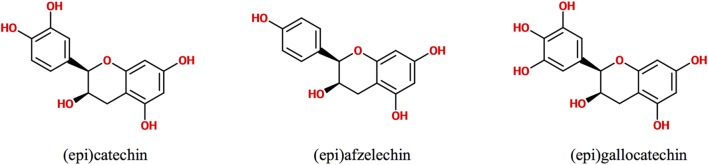
**Chemical structure of flavanol units conforming proanthocyanidins**.

**Figure 2:**
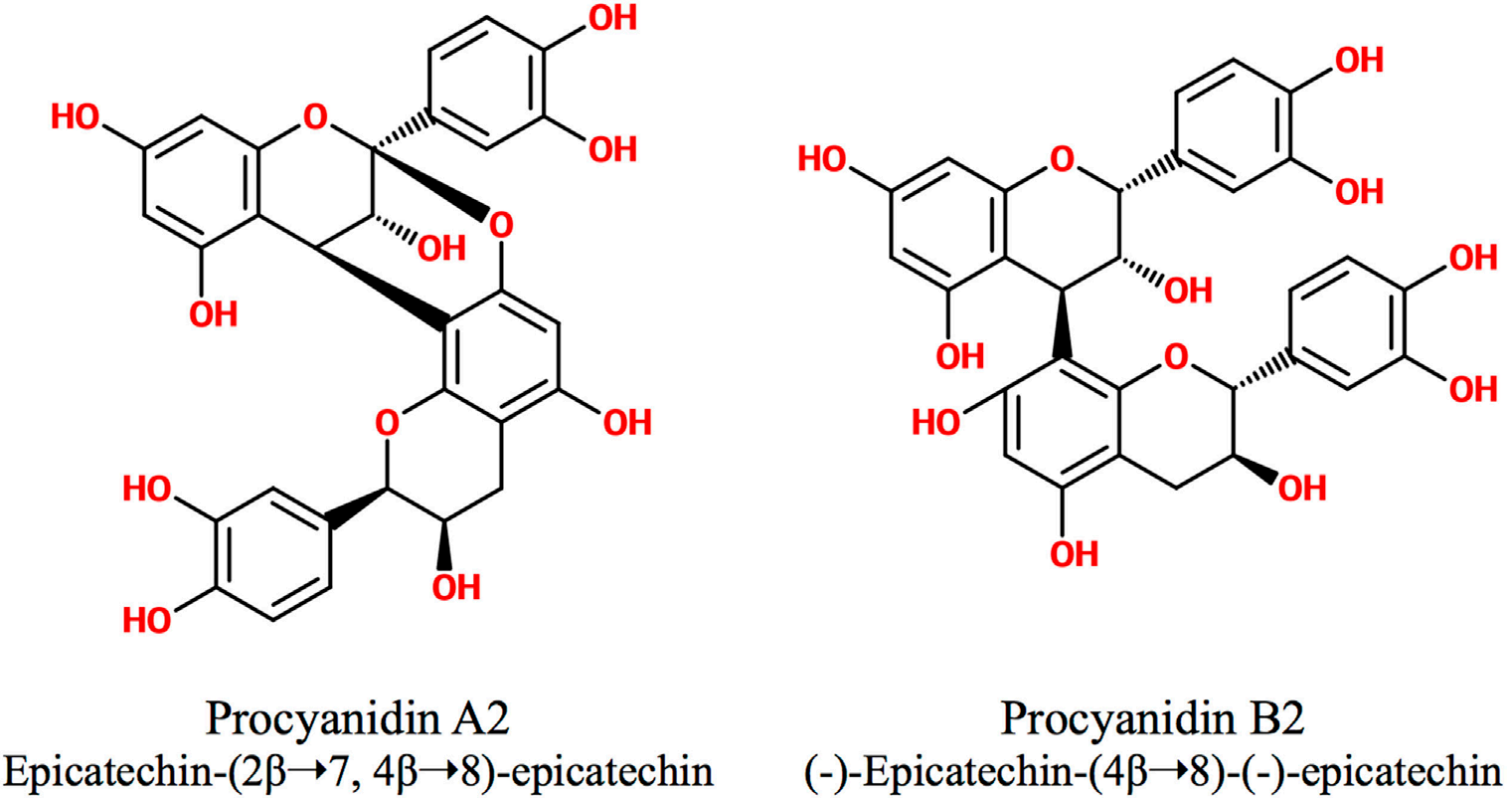
**Type-A and type-B proanthocyanidins**.

Polyphenols are secondary metabolites synthesized by plants, which are implicated in their protection against microbial pathogens, predators, ultraviolet radiation, and adverse conditions of nutrition and growth (1, 2). They are classified as non-flavonoids and flavonoids, being these latter the major group of phytochemicals present in the human diet. The flavonoid chemical structure is characterized by two aromatic rings connected by a three-carbon bridge (C6–C3–C6) (3, 4). The main subclasses of flavonoids are flavones, flavonols, flavan-3-ols, isoflavones, flavanones, and anthocyanidins (3). The flavan-3-ols (also called flavanols) are the most complex as they include not only simple monomers but also oligomeric and polymeric proanthocyanidins (PACs), known as condensed tannins (3). Accordingly, PAC size is variable and depends on their degree of polymerization (DP), i.e., the amount of monomers of flavan-3-ols incorporated in the molecule. The DP commonly varies between 3 and 11 but can even reach up 50 units or more. For example, the average DP of black gooseberry (*Ribes nigrum*) PACs is about 48, whereas in wine and beer PACs, it is only 7 and 2, respectively (3, 5). PACs are abundant in many foods and beverages like seeds, barks, fruits, red wine, cider, tea, cocoa, and beer, where they contribute to their bitter taste and astringency (4, 5). Depending on the units of flavanols involved in their structure, PACs are subdivided in different classes. The most abundant consist exclusively of (epi)catechin units and are called procyanidins (PCs), while the less common containing (epi)afzelechin or (epi)gallocatechin subunits are named propelargonidins and prodelphinidins, respectively (3, 4, 6, 7) (Figure 1). PACs may also be classified as type-A or -B according to the interflavanol linkage; type-B PACs are found in a greater abundance and have only one C–C interflavan bond, while type-A are less common and are characterized by an additional ether linkage (7) (Figure 2). Type-B PACs are present in fruits (apples, grapes, pears), legumes, cereals (barley, sorghum), cocoa, and their derived foodstuffs (wine, cider, beer, etc.), while A-type are found in cranberry, cinnamon, apricots, and avocado, among others; some foodstuffs have mixed type-A and -B PACs (7–10).

The beneficial impact of dietary PACs on the risk of cancer (5, 11, 12), cardiovascular diseases (5, 13, 14), and diabetes (12, 15, 16) is supported by a large number of *in vitro*, animal, clinical, and epidemiological studies. These health-promoting effects suggest that PACs may be absorbed by the intestinal mucosa. However, evidence indicates that only monomers, dimers and, eventually, trimers of flavan-3-ols are absorbable, while oligomers and polymers of higher DP remain unabsorbed and accumulate in the gut lumen. Non-absorbed PACs reach the colon where they are metabolized by the microbiota, producing low molecular weight (LMW) compounds (4, 7, 17–19) that may be absorbed into the circulation. Therefore, the beneficial effect of PACs on human health may be attributed not only to the circulating monomers or dimers of flavanols but also to the microbiota-derived PAC metabolites present in the bloodstream (7). Additionally, PACs and/or their bacterial metabolites may exert health benefits directly in the gastrointestinal (GI) tract through their antioxidant, antiinflammatory, antibacterial, and antiproliferative properties (12).

**Figure 3:**
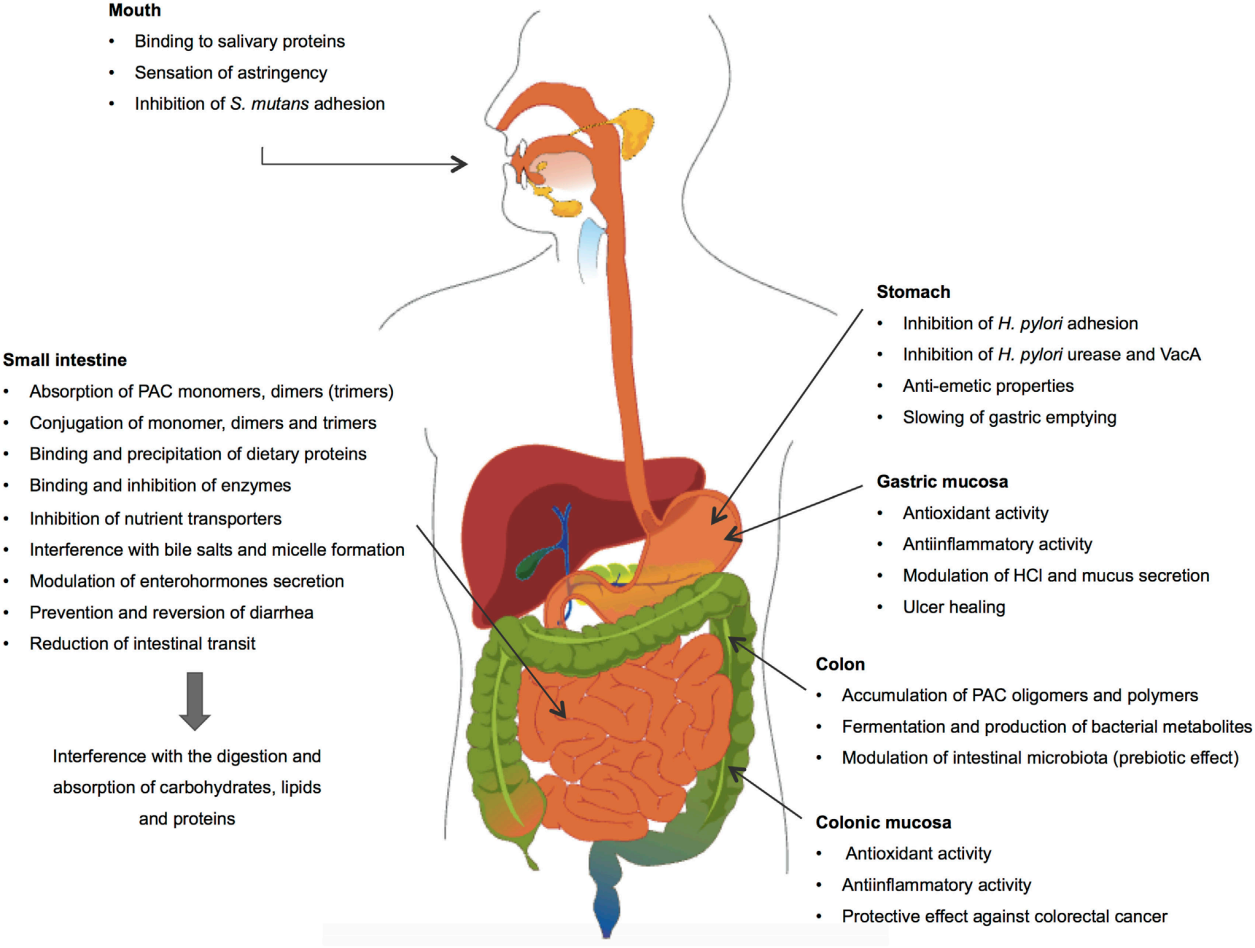
**Interactions between dietary proanthocyanidins and the gastrointestinal tract**.

The present review describes how dietary PACs impact the main physiological processes occurring in the GI tract and exerts protective properties in pathological conditions (Figure 3). More particularly, it addresses the fate of PACs in the different compartments of the GI tract, describing their absorption and metabolism, and their ability to interfere with the processes of digestion and absorption of macronutrients in the intestine and to modulate digestive hormone secretion, hydroelectrolytic epithelial transport, and GI motility. In parallel, this review also describes the protective effects displayed by dietary PACs including the attenuation of enteropathogen deleterious activities and gastric and colonic inflammatory processes. Finally, it addresses their prebiotic effect in the colon, their transformation by the microbiota, and their role in decreasing the risk of colorectal cancer.

## PACs in the Mouth

Dietary PACs have been shown to bind and precipitate salivary proteins containing high-proline contents, a phenomenon that constitutes the physiologic base of astringency perception after consuming PAC-containing foodstuffs (20). Catechins were detected in saliva for up to 60 min after using a mouth rinsing product containing green tea extract (5 mg/ml) (21); the persistence of these compounds in saliva could favor their antimicrobial activity against some oral bacteria. More particularly, cranberry PACs have been shown to inhibit the adhesion of the cariogenic bacteria *Streptococcus mutans to* oral epithelial cells, thus preventing the formation of pathogen biofilm in the tooth surface (22, 23). On the other hand, PACs containing galloyl moieties have been recently proposed as a useful tool in restorative and reparative dentistry, due to their ability to act as dentin biomodifiers (24).

## PACs in the Stomach

### Protective Effect against *Helicobacter pylori* (*H. pylori*)

*Helicobacter pylori* is a helix-shaped, microaerophilic, Gram-negative, flagellated bacterium that specifically colonizes the human gastric mucosa. It is considered as the most widespread chronic bacterial pathogen in the world, infecting more than 50% of the human population. Although over 80% of the infected individuals remain asymptomatic, *H. pylori* is implicated as an etiologic factor in the development of a variety of GI diseases including gastroduodenal ulcers and gastric adenocarcinoma and lymphoma. Accordingly, this pathogen is classified as a class I carcinogen (25–27). *H. pylori* expresses several virulence factors involved in the initial colonization of the gastric mucosa by the bacteria and in its persistence in the stomach. These factors include (1) adhesins, bacterial surface proteins allowing the adherence of this agent to the gastric epithelial cells, (2) an urease activity that releases ammonia from urea hydrolysis, allowing proton neutralization and the survival of the bacteria in the acidic gastric environment, (3) the vacuolating cytotoxin A (VacA) that alters the mitochondrial function of the epithelial cells and promotes their vacuolization and subsequent apoptosis, in addition to influencing host tolerance by suppressing T cell activation, and (4) the cag pathogenicity island encoding a type IV secretion system, which is involved in the development of inflammatory processes in the mucosa (27–29).

The administration of PACs-rich beverages decreases *H. pylori* colonization in humans. A prospective, randomized, double-blind, placebo-controlled trial conducted in China in 189 adult patients colonized by *H. pylori* provided some clinical support for the anti-*H. pylori* effect of cranberry juice that contains large amounts of A-type PACs (30, 31). The participants were assigned to receive 250 ml of cranberry juice or placebo twice daily for 90 days. *H. pylori* colonization was determined by ^13^C-urea breath test after 35 and 90 days of treatment. The eradication of the pathogen was reported in 14.4% of the treated subjects compared with 5.3% in the placebo group. Similar results were reported in a clinical trial carried out in 295 Chilean children colonized by *H. pylori*. The intake of 200 ml cranberry juice per day for 3 weeks induced the eradication of the pathogen in 16.9% of the treated children vs 1.5% in the placebo group. Interestingly, the coadministration of cranberry juice with the probiotic, *L. johnsonii* NCC533, eradicated the bacteria in 22.9% of the children (32). The mechanisms associated with the anti-*H. pylori* activity of cranberry PACs include their ability to inhibit urease activity and prevent bacterial adhesion as well as VacA-induced mucosal damage (Table 1). High molecular mass, non-dialyzable constituents of cranberry juice, subsequently characterized as PACs, have been shown to inhibit the adhesion of clinically isolated *H. pylori* strains to AGS gastric cell line. As no cross-resistance was detected between the non-dialyzable material and metronidazole, it was suggested that cranberry preparations could improve *H. pylori* eradication in untreated patients as well as in those under pharmacological treatment (33). *In vitro* studies consistently indicate that high molecular weight (HMW) components from cranberry inhibit the sialyllactose-specific (S fimbriae) adhesion of *H. pylori* to immobilized human mucus, erythrocytes, and cultured gastric epithelial cells (34, 35). This anti-adhesion effect was not restricted to cranberry PACs and was also reported with dietary PACs from different sources. Pycnogenol^®^, a standardized concentrate elaborated from French maritime pine bark and which contains PAC from dimers (mainly type B) to polymers (up to 12 monomeric units of flavanol) (36, 37), inhibited concentration-dependently the adhesion of clinical strains of *H. pylori* to AGS cells (38). A root extract from *Pelargonium sidoides*, rich in polymeric PACs (39), has also been shown to prevent *H. pylori* adhesion to intact human stomach tissue (40–42).

**Table 1:** **Anti-*Helicobacter pylori* (*H. pylori*) effects of proanthocyanidins**.

Effects	Extract-compound	Model	Reference
Decrease of *H. pylori* colonization	250 ml *Vaccinium macrocarpon* juice, twice a day, for 35 and 90 days	189 human adults	(30)

Decrease of *H. pylori* colonization	200 ml *V. macrocarpon* juice per day for 3 weeks	295 children	(32)

Inhibition of the adhesion of *H. pylori* strains to epithelia	High molecular mass non-dialyzable constituents of *V. macrocarpon* juice	AGS cells	(33)
	
	High molecular mass constituents from *V. macrocarpon* juice	Human gastric mucus HT-29 cells	(34, 35)
	
	Pycnogenol^®^, a standardized PACs (B-type, C4–C8 bonds) extract from the French maritime pine bark	AGS cells	(38)
	
	*Pelargonium sidoides* root extract containing mainly polymeric PACs	*In situ* anti-adhesion assay to intact human stomach tissue	(40–42)

Inhibition of urease activity	*Eucalyptus grandis* (Myrtaceae) stem bark extract	Urease from three clinical isolates *H. pylori* strains	(43)
	
	An *Malus domestica* peel polyphenol extract (APPE), containing epicatechin-derived procyanidins (PCs) B1, B2, and C1 APPE-derived high molecular weight (HMW) extract APPE-derived low molecular weight (LMW) extract	*H. pylori* (ATCC 43504) and Jack bean ureases	(44)
	
	*Peumus boldus* aqueous extract (BAE), containing catechin-derived PCs B3 and C2 Subfraction of BAE according to the degree of polymerization content	*H. pylori* (ATCC 43504) and Jack bean ureases	(45)
	
	B-type PCs (catechin dimers)	Molecular docking analysis of urease inhibition	(48)

Vacuolating cytotoxin A (VacA) inhibition	*Humulus lupulus* bract extract (HBT) containing HMW polymerized catechins HBT-derived HMW extract HBT-derived LMW extract	Mouse model of experimental VacA infection	(46)
	
	Red wine and green tea mixture	Mouse model of experimental VacA and *H. pylori* infection	(47)

Proanthocyanidins can also interfere with *H. pylori* by inhibiting its urease activity and/or by inactivating VacA. For example, a methanol extract of *Eucalyptus grandis* (Myrtaceae) bark was shown to inhibit the urease of clinical strains of *H. pylori* in a concentration-dependent manner. This effect was attributed to the tannins and triterpene saponins present in the extract (43). Some studies suggest that PAC DP is a determining factor for urease inhibition or their protective effects on VacA-induced mucosal damage. LMW PAC fraction (mean DP of 3) derived from apple peel exhibited an urease inhibition fourfold lower than the high HMW fraction (mean DP of 9.5) (44). More recently, an aqueous extract of *Peumus boldus* Mol. (Monimiaceae) rich in catechin-derived PCs B3 and C2 was also shown to inhibit *H. pylori* urease (45). The separation of the extract components according to their molecular weight revealed that the higher the DP, the greater the urease inhibition. Interestingly, the catechin-derived PCs (B3 and C2) were more effective than the epicatechin-derived PCs B2 and C1 in inhibiting the urease, suggesting that not only DP is important but also the chemical nature of the monomer bound to C-4 in the PC structure (45). A structure–activity relationship between PAC DP and their ability to inactivate VacA cytotoxin has also been established with hops extracts (46). Accordingly, the administration of red wine or green tea mixture to *H. pylori*-infected mice significantly prevented the development of gastritis, limiting the localization of the pathogen and VacA toxin on the surface of the gastric epithelium (47).

On the other hand, exciting advances in the knowledge of the interactions between PCs and target molecules are emerging from molecular docking studies. Such studies, for example, revealed that B-type PCs (catechin dimers) inhibit urease because, according to their docking scores (−6.9 kcal/mol), they fit the binding pocket of the bacterial enzyme, being these interactions energetically favorable (48). Then, molecular docking could contribute to elucidate the interactions between PCs and other cellular or bacterial targets of interest for the protection of the gastric mucosa against *H. pylori*.

### Protective Effect against Gastric Inflammation

The gastroprotective properties of PAC extracts have been widely studied using different animal models of gastric inflammation induced by ethanol, non-steroidal antiinflammatory drugs (NSAIDs), pylorus ligature, or restrain stress (Table 2). Synthetic PAC oligomers, more particularly those bigger than tetramers, protected the gastric mucosa against ethanol-induced damage, due to their ability to scavenge free radicals and, therefore, prevented the appearance of oxidative damage (49). Such effect could also be due to the protein-binding ability of the oligomers, which would allow them to form a protective layer coating the gastric mucosa. In another study, the administration of a single oral dose of Hawthorn berries extract (a mixture of *Crataegus monogyna* and *C. oxyacantha* containing 0.44% PACs) attenuated the intensity of ethanol-induced gastric lesions in rats, an effect similar to that observed with the administration of ranitidine (50). Considering the low proportion of PACs in this extract, it is improbable that these molecules were responsible for this protective effect; however, it is important to mention that Hawthorn berries contain oligomeric PACs and more B-type than A-type (51).

**Table 2:** **Gastroprotective effects of proanthocyanidins**.

Extract-compound	Model of gastric mucosal lesions	Effects	Reference
Synthetic proanthocyanidin oligomers Single dose (200 mg/kg), p.o.	(HCl)/ethanol	↓ gastric lesion	(49)

Gravinol S extract 0.002, 0.02, 0.2, and 1% in drinking water for 2 weeks	Water-immersion restraint	↓ gastric lesion ↓ MPO activity ↑ SOD activity ↓ gastrin and somatostatin levels ↑ PGE_2_ levels	(64)

*Viburnum opulus* extract 25, 50, or 75 mg/kg/day, p.o. For 3 days	Water-immersion restraint	↓ gastric lesion ↑ NO expression ↑ SOD, CAT, and GSPHpx activities	(65)

*Guazuma ulmifolia* extract 500, 250, and 125 mg/kg/day, p.o. For 2 days	NSAID	↓ gastric lesion ↓ neutrophil infiltration ↓ lipid peroxidation ↑ SOD and GSHpx activities No effect on PGE_2_ levels	(54)

*Hippophaë rhamnoides* L. extract 50, 100, and 150 mg/kg/day, p.o. For 7 or 14 days after acetic acid	Acetic acid	Acceleration of mucosa repair ↑ plasmatic EGF ↑ EGFR and PCNA expressions in the gastric ulcer tissues	(63)

*Hancornia speciosa* extract Single dose (250, 500, or 1,000 mg/kg), p.o.	(HCl)/ethanol NSAID	↓ gastric lesion	(57)

*H. speciosa* extract Single dose (500 mg/kg), p.o.	Hypothermic restraint	↓ gastric lesion	(57)
	
	Pylorus ligation	↓ gastric lesion ↑ pH, without changing the gastric volume	(57)

*H. speciosa* extract 500 mg/kg/day, p.o. For 7 or 14 days after acetic acid	Acetic acid	Acceleration of mucosa repair	(57)

*Cecropia glazioui* Sneth extract butanolic fraction Single dose (0.05–0.5 g/kg), p.o.	(HCl)/ethanol NSAID Hypothermic restraint	↓ gastric lesion	(60)

*C. glazioui* Sneth extract butanolic fraction Single dose (0.5–2.0 g/kg), i.d.	Pylorus ligation	↓ gastric lesion ↓ acid secretion, volume, and acidity	(60)

*Crataegus monogyna* and *Cratageus oxycantha* extract Single dose (50–200 mg/kg), p.o.	(HCl)/ethanol	↓ gastric lesion	(50)

*Curatella americana* L. hydroalcoholic extract Single dose (100, 250, 500, or 1,000 mg/kg), p.o.	NSAID	↓ gastric lesion	(58)
	
	(HCl)/ethanol	↓ gastric lesion ↓ gastrin hormone levels ↑ somatostatin hormone levels ↑ PGE_2_ levels ↑ mucus production	(58)

*C. americana* L. hydroalcoholic extract Single dose (500 mg/kg), p.o.	Hypothermic restraint	↓ gastric lesion	(58)
	
	Pylorus ligation	↓ gastric lesion ↑ gastric pH	(58)

*C. americana* L. hydroalcoholic extract 500 mg/kg/day, p.o. For 14 days after acetic acid	Acetic acid	Acceleration of mucosa repair	(58)

*Byrsonima intermedia* extract Single dose (250, 500, or 1,000 mg/kg), p.o.	(HCl)/ethanol, NSAID	↓ gastric lesion	(61)

*B. intermedia* extract Single dose (500 mg/kg), p.o.	Pylorus ligation	↓ gastric lesion ↑ gastric volume, without changing the pH	(61)

*B. intermedia* extract 500 mg/kg/day, p.o. For 7 or 14 days after acetic acid	Acetic acid	Acceleration of mucosa repair	(61)

*Vitis vinifera* seed proanthocyanidin extracts 100 and 300 mg/kg/day, p.o. For 6 days	NSAID	↓ gastric lesion ↑ glutathione levels No effect in PGE_2_ levels	(55)

*CAT, catalase; EGF, epidermal growth factor; EGFR, epidermal growth factor receptor; GSPHpx, gluthatione peroxidase; i.d. intraduodenally; MPO, myeloperoxidase; NO, nitric oxide; NSAID, non-steroidal antiinflammatory drug; PCNA, proliferating cell nuclear antigen; p.o. orally; SOD, superoxide dismutase; PGE_2_, prostaglandin E_2_*.

Proanthocyanidins were also protective in gastric damage induced by NSAIDs. Although NSAIDs are widely used for their antipyretic, analgesic, and antiinflammatory properties, their administration is frequently associated with adverse effects mainly affecting the GI mucosa (52, 53). NSAIDs and proton pump inhibitors (like omeprazole and lansoprazole) are frequently coprescribed to minimize NSAID-related adverse effects and the enteropathy induced by such combination, though common, is often clinically silent. Thus, the lesions induced by these drugs in the GI tract could be of considerable clinical importance. Accordingly, bioactive compounds, like PACs, arise as an alternative approach for the management of the adverse effects associated with NSAID therapies (53). The oral intake of PAC extracts from *Guazuma ulmifolia* (*Sterculiaceae*) or grape seed for 2–6 days before diclofenac or indomethacin administration in rats, prevented in a dose-dependent manner the development of gastric mucosal damage and attenuated intestinal injury (54–56). These extracts decreased the area of ulceration induced by indomethacin in the stomach by decreasing lipid peroxidation in the mucosa and by increasing the superoxide dismutase and glutathione peroxidase activities as well as the glutathione levels (54). The *G. ulmifolia* extract was also shown to prevent neutrophil infiltration (reflected by a lower myeloperoxidase activity) in the mucosa (54).

Proanthocyanidin extracts from medicinal plants including Mangaba (*Hancornia speciosa*) (containing LMW PACs), *Curatella americana* L. (Dilleneaceae) (containing oligomeric and polymeric PACs), *Cecropia glazioui* Sneth (Cecropiaceae) (that contains 22% of PACs B2, B3, B5, and C1), and *Byrsonima intermedia* (containing oligomeric PACs, phenolic acids, and catechin derivatives and flavonoids) have also been evaluated (57–61). The oral administration of a single dose of each extract decreased the gastric lesions induced by indomethacin or piroxicam, hypothermic restraint stress, ethanol, or pylorus ligature, in the same extent that cimetidine, ranitidine, or lansoprazole (57, 58, 60, 61). Interestingly, the extraction step seems to be critical to determine the antiulcer effects of the bioactive compounds; no significant protective effects were observed when plant infusions were used (57, 58).

Another mechanism by which PACs may protect the gastric mucosa is through the modulation of HCl secretion. Plant extracts decreased the secretion of HCl by gastric parietal cells when administered intraduodenally to pylorus-ligated mice (57, 58, 60, 61). The *C. glazioui* extract reversed the histamine or bethanechol-induced acid secretion to basal values, indicating it inhibits the proton pump. This antisecretory effect was comparable to that observed with the histamine H_2_ receptor-antagonist, ranitidine (60). It is possible that this effect relies on the type-B2 PACs since these molecules displayed the highest inhibitory activity against the gastric H^+^, K^+^-ATPase *in vitro*, compared with the other type of PACs isolated from *C. glazioui* (like B3, B5, and C1) (60).

The PAC antiulcer properties are also related to their ability to stimulate mucus synthesis and secretion (62), and to their mucosal repair activity. Indeed, they were shown to accelerate the healing of gastric ulcer induced by acetic acid administration (57, 58, 61). It is probable that nitric oxide synthase (NOS), sulfhydryl compounds (SH), and TRPV-vanilloid receptors were involved in this phenomenon, as pretreatment with NOS inhibitor (L-NAME), SH-blocker (NEM), or TRPV receptor inhibitor (ruthenium red) blocked the PACs protection against ethanol-induced gastric damage (57, 58, 61). The increased expression of key molecules implicated in the restitution of the gastric epithelium during chronic gastroduodenal ulcers arises as another mechanism of protection exerted by PACs. For example, a sea buckthorn extract containing 96.5% PACs was shown to decrease the ulcer index in acetic acid-induced gastric lesions, in association with increased plasma concentrations of epidermal growth factor (EGF) and higher expression of EGF receptor and proliferating cell nuclear antigen in the gastric mucosa (63). These molecules are considered as crucial for the ulcer healing process and are implicated in epithelial restitution and gland reconstruction. The effect of PACs on trefoil peptides, also involved in the epithelial restitution process, has not been studied.

On the other hand, PACs could also exert their protective effects through endocrine and neural mechanisms. A *C. americana* extract, for example, exhibited ulcer healing properties by increasing the mucosal levels of prostaglandin E_2_ and somatostatin and decreasing those of gastrin (58). Gravinol S containing 89.3% PAC (25.2% dimers–pentamers, 74.8% oligomers) from grape seeds, when administered *ad libitum* for 2 weeks, prevented gastric mucosal damage induced by water immersion restraint stress in rats. The authors proposed that PACs inhibit gastrin secretion by G cells and subsequently that of histamine and somatostatin, in addition to increase prostaglandin levels and superoxide dismutase activity in the gastric mucosa (64). Using the same animal model, a single dose of *Viburnum opulus* (*Caprifoliaceae*) PAC extract attenuated the gastroduodenal lesions. This effect was abrogated by capsaicin pretreatment, suggesting the implication of the nervous system. Moreover, the administration of this extract was also associated with the stimulation of the nitric oxide system, the increased resistance of the mucus layer, and the stimulation of mucosal superoxide dismutase and catalase activities (65).

### Effect on GI Hormone Secretion and Gastric Emptying

A few studies have evaluated the impact of PAC intake on the release of digestive hormones, some of them in relation to GI motility. González-Abuín et al. showed that rats fed a cafeteria diet exhibited a lower density of enteroendocrine cells in the intestinal epithelium and plasma concentrations of active glucagon-like peptide-1 (GLP-1), and that these alterations were prevented with a grape seed PC extract (66). Since GLP-1 is an incretin hormone participating in the regulation of food intake and insulin secretion, it is possible that dietary PACs are beneficial for the control of energetic metabolism in humans. There is no doubt that it is a promising field of research. Serrano et al. showed that the administration of a grape seed proanthocyanidin extract (423 mg phenolics/kg body weight) to rats increased the portal concentrations of active GLP-1 and ghrelin and decreased those of cholecystokinin. These findings were accompanied by a delayed gastric emptying and lower food intake in the treated animals (67). In another study, Ko et al. evaluated the effect of a whole grape juice (with skin and seeds) in rats treated with cisplatin, a chemotherapeutic drug known to provoke acute GI disorders. Cisplatin treatment decreased significantly the rate of gastric emptying compared with the control group, and pretreatment with grape juice (10 ml/kg body weight) prevented this disturbance (68).

### Antiemetic Properties

Miller et al. evaluated the effect of an extract of *Croton palanostigma* (100 mg/g of PACs) in the treatment of emesis induced by the administration of morphine-6-glucuronide in adult ferrets. The extract reduced by 77% the morphine-induced vomiting and retching, suggesting that it could suppress the activation of sensory afferent nerve implicated in the emetic reflex (69). According to these observations, Li et al. reported that a grape seed proanthocyanidin extract inhibits in a non-competitive manner the 5-hydroxytryptamine-3 receptors involved in the initiation and coordination of the vomiting reflex, in NCB-20 neuroblastoma cells (70). Taking together, these results suggest that PAC-containing extracts or foodstuffs could be used as a complementary medicine for the management of nausea/emesis, without the side effect usually associated with cannabinoid-based antiemetic agents.

## Absorption of Flavan-3-ols and PACs

Gastric degradation of PC oligomers has been observed after their incubation in simulated gastric conditions (pH2) *in vitro* (71). However, these observations were not confirmed by Rios et al., who investigated the gastric stability of PCs in 6 human volunteers after ingestion of cocoa drink containing 733 mg PC polymers and 351 mg flavanol monomers. Gastric samples were collected through a nasogastric tube every 10 min until total gastric emptying (52–60 min), and PCs were quantified. No degradation of these molecules was detected, suggesting their great stability in the stomach environment, and that most of them reach the small intestine intact (72).

Studies in *in vitro* models or carried out both in humans and animals generally report that PAC monomers, dimers, and eventually trimers may be absorbed in the intestine, while larger polymers remain unabsorbed and accumulate in the gut lumen (73–75). Such absorption is directly proportional to the PACs luminal concentrations, as shown in rats by using intestinal perfusion of different (+)-catechin concentrations (1–100mM) (76). In humans, catechins have been detected in plasma as early as 30 min after drinking green or black tea (77). The intestinal absorption of flavan-3-ols has also been studied in patients with ileostomy after the ingestion of 200 mg of a green tea extract. About 40% of the flavan-3-ols administered were recovered in the ileostomy bag, confirming that substantial amounts of these molecules are absorbed in the small intestine (78). In this study, sulfate, glucuronide, and methylated conjugated metabolites were identified in plasma, all derived from (epi)catechin or (epi)gallocatechin, representing 47 and 26%, respectively, of the parent compounds present in the extract. Data concerning PAC dimers are more controversial, and some authors could not detect any absorption of these compounds in the intestine. Donovan et al., for example, showed that the PAC dimers B1, B2, and B3 were not absorbed in rats neither hydrolyzed to their corresponding monomers in the intestine (79). The absorption of trimers and oligomers is also controversial. Tsang et al. provide evidence that PAC oligomers were not depolymerized to monomers to any extent after ingestion of a grape seed extract (80). In this study, only catechin glucuronides and methylated glucuronide metabolites were detected in plasma as well as in the kidneys and liver. These metabolites were also found in urine with sulfate metabolites and low amounts of the dimers B1, B2, B3, and B4, and the trimer C2. In opposition with these results, Shoji et al. detected oligomers with a mean DP of 2–5 in rat plasma 2 h after the administration of apple PACs with the same DP (81). In another study, 11% of ingested PACs from grape seed extract were recovered in feces, and 71% of them were tetramers to hexamers, suggesting that PACs with more than 3 subunits are more resistant to degradation and accumulate in the colonic lumen when they may be detected in high levels (82). These observations were confirmed by Jimenez-Ramsey et al. and Terrill et al. using ^14^C-labeled polymeric PACs in chickens and sheep; most of these molecules were not absorbed in the intestine of the animals and were widely recovered in their feces (83, 84). Similar findings were reported in pigs fed grape seed PACs (dimers–pentamers); those were not completely absorbed and remained transiting in the gut lumen for at least 72 h before their fecal excretion (85). In another study in ileostomized subjects, 90% of the PACs consumed as apple juice were found in the ileostomy effluent; however, the DP of the recovered PACs was reduced to 3.4, in comparison to the initial DP of 5.7 in the apple juice. These results, therefore, suggest that part of the oligomeric procyanidins were cleaved into smaller units that, eventually, were absorbed (86).

From these studies it may be concluded that dietary PACs are not affected by their passage across the stomach and that in the small intestine, only monomers, dimers, and eventually trimers may be absorbed to some extent, while larger oligomers and polymers remain in the lumen and accumulate in the colon.

## Intestinal Metabolism and Bioavailability of Flavan-3-ols and PACs

Factors other than DP also affect PAC bioavailability. The fact that flavanols are frequently acylated, especially by gallic acid, reduces their absorption (87) even whether galloylation does not dramatically influence PACs bioavailability as glycosylation with other polyphenols (88). The initial step in the intestinal absorption of dietary flavonoid glucosides is their deglycosylation, which would occur in the enterocyte brush-border membrane through the lactase–phlorizin hydrolase and beta-glycosidase enzymes (89). This event releases a free aglycone that can then enter into epithelial cells either passively or by facilitated diffusion (90). However, flavan-3-ols are the only subclass of flavonoids present in unglycosylated forms in plants, being found naturally as aglycones (91). Therefore, the flavan3-ols are absorbed by the enterocytes without any deconjugation or hydrolysis (88). On the other hand, flavonoids including flavonol-3-ols are generally recognized as xenobiotics by the intestinal detoxification system (92). Accordingly, they may be subjected to Phase II biotransformation (conjugation) in the enterocytes and posteriorly in the hepatocytes, resulting in a series of water-soluble conjugated metabolites including methyl, glucuronide, and sulfate derivatives (93). The role of the small intestine in the glucuronidation and methylation of catechins and that of the liver in their sulfation, methylation, and biliary excretion has been described in rats by Donovan et al. (76). Conjugated compounds are released into the systemic circulation for their further distribution to the body organs and excretion in urine, or are exported into the bile to come back into the intestinal lumen, reaching the colon where they may be metabolized by the microbiota or reabsorbed, leading to an enterohepatic cycling. After the administration of 500 ml of green tea containing 648 µmol of flavan-3-ols in ileostomized subjects, Stalmach et al. quantified the conjugated forms of flavanols present in plasma, urine, and ileal effluents. Sixteen metabolites were detected in plasma and 18 in urine. In the ileal effluents, 70% of the ingested flavan-3-ols were present in their native form and 23 metabolites corresponding to conjugated forms resecreted into the intestinal lumen were detected, mainly sulfate and methyl-sulfate derivatives from epicatechin and epigallocatechin (94). Flavanols seem to be present in plasma as free flavanols as well as sulfate and glucuronide forms, according the type of flavanol (95). In fact, the methylated metabolites of catechin, epicatechin, and epicatechin gallate predominate over the original unmethylated forms in plasma (96). Lee et al. determined flavanol conjugates in plasma after ingestion of green tea in humans. (−)-Epigallocatechin-3-gallate was mainly detected as sulfate conjugate (65%), followed by the free form (20%) and glucuronide form (15%), while (−)-epigallocatechin was mostly found in the glucuronide form (60%) followed by the sulfate form (30%) and the unconjugated (10%). (−)-Epicatechin was exclusively found in the conjugated form, with approximately two-thirds sulfate and one-third glucuronide (97).

As previously stated, most of the ingested PACs remain unabsorbed in the small intestine and accumulate in the colon (98) where they are degraded by the colonic microbiota in low molecular weight aromatic acids, which differ according to their hydroxylation profile and the length of their aliphatic side chain (18). These microbial metabolites are absorbed in the colon and may be also conjugated by the colonocytes or in liver, resulting in glucuronide, methyl, glycine, and sulfate derivatives (99).

## Effects of PACs in the Digestion and Absorption of Nutrients

The intestinal lumen is the main site of interactions between dietary PACs and nutrients and enzymes. These interactions occur thanks to the chemical structure of PACs and their numerous hydroxyl groups suitable for forming non-specific complexes with proteins, resulting in their precipitation (100). This event occurs preferentially at pH values near the protein isoelectric point. Hagerman and Butler have observed that PAC affinity for proteins was inversely proportional to protein size and depended on their proline content (101). The interaction between PACs and proteins constitutes the base of the tanning process that transforms animal hides into leather through the complexation of skin collagen and the oral sensation of astringency through the complexation of salivary proline-rich glycoproteins. Accordingly, PACs may also interact with the pancreatic enzymes released in the intestinal lumen and with brush-border enzymes and nutrient transporters, thus affecting nutrient bioavailability (102, 103).

### Effects on the Digestion and Absorption of Dietary Carbohydrates

Starch constitutes the main source of carbohydrates and energy in the occidental diet, although the disaccharides sucrose and lactose and the monosaccharides glucose and fructose are also present (104). Starch is digested in the intestinal lumen by pancreatic α-amylase and the resulting disaccharides, trisaccharides, and limit dextrin are subsequently digested by the brush-border disaccharidases, maltase-glucoamylase and saccharase-isomaltase, while lactose is hydrolyzed by the lactase-phloridzin hydrolase. The resulting monosaccharides (glucose, galactose, and fructose) are absorbed into the enterocytes through active [sodium–glucose cotransporter 1 (SGLT1)] and facilitated [glucose transporter (GLUT5 and GLUT2)] apical transporters. Dietary polyphenols, including PACs, may delay carbohydrate digestion and reduce postprandial glucose absorption, which represents an alternative approach for diabetes prevention and management (15, 102).

#### Inhibition of Enzymes Involved in Carbohydrate Digestion

A number of *in vitro* studies have evaluated the inhibitory activity of different PACs against α-amylase and disaccharidases (Table 3). Fractionated polymeric and oligomeric PACs from peel persimmon inhibited α-amylase and α-glucosidase *in vitro*. PAC polymers had higher inhibitory activity against α-amylase than oligomers, while the opposite was observed against α-glucosidase. These results suggest that PAC DP is related to the inhibition of these enzymes, and that the oligomers have probably a greater potential than polymers for diabetes prevention or management (105). In another *in vitro* study, aqueous and alcoholic grape seed extracts were shown to inhibit α-amylase dose dependently, being more elevated the inhibition with the ethanolic extract (around 75%) than with the aqueous (around 52%) at the same concentration (106). The inhibitory effect of four aqueous extracts from different species of cinnamon barks (condensed tannins ranging between 0.12 and 0.15 g catechin equivalent/g extract) against α-amylase, maltase, and sucrase activities was determined *in vitro*. Thai cinnamon was the most potent maltase inhibitor and Ceylon cinnamon the most efficient in suppressing sucrase and α-amylase. When combined with acarbose (a recognized α-glucosidase inhibitor), all the extracts displayed an additive inhibition against α-amylase, while only the Chinese, Ceylon, and Thai cinnamon extracts (CEs) showed an additive inhibition against sucrase and maltase (107). Interestingly, these results suggest that PACs from the same botanical species may differ in their PAC composition depending on the geographical origin of the plant, probably due to differences in geo-climatic conditions of culture. Pycnogenol^®^ was also shown to inhibit baker’s yeast α-glucosidase more efficiently than green tea extract and acarbose. This could explain the glucose-lowering effects reported with this product in clinical trials with diabetic patients (108, 109). PAC fractions purified from peanut skin also showed an inhibitory activity against maltase and sucrase at a concentration of 1 mg/ml. The strongest maltase inhibition was exerted by the trimeric PAC epicatechin-(2β → O → 7,4β → 8)-[catechin-(6 → 4β)]-epicatechin, while the strongest sucrase inhibition was exhibited by another trimeric PAC, epicatechin-(4β → 8)-epicatechin-(2β → O → 7,4β → 8)-catechin. The inhibitory activity of both compounds was lower than acarbose but higher than that of dimeric PACs (110). Barrett et al. studied the effect of condensed tannins from cranberry, grapes, and cocoa extracts against α-amylase and glucoamylase *in vitro*, using different tannin:enzyme ratios (0.01:1 to1:1) (103). Cocoa, grape, and cranberry tannins exerted the highest inhibition of α-amylase and glucoamylase at 1:1 ratio (14, 28, and 55%, respectively, for α-amylase and 23, 55, and 41% respectively for glucoamylase). Cocoa, cranberry, and grape tannins also reduced glucoamylase activity in approximately 20% at the 0.01:1 ratio. Grape and cranberry increased their inhibitory activity against the enzyme at 1:1 ratio (55 and 41%, respectively). Accordingly, in this study, the inhibitory effect was strongly dependent on the tannin concentration and HMW tannins (like those present in cranberries) have greater inhibitory capacity than LMW tannins (like those present in cocoa). Similar findings were also described by Tsujita et al. with different fractions of peanut seed skin (111) and almond seed skin (112).

**Table 3:** **Impact of proanthocyanidins on enzymes involved in carbohydrate digestion**.

Type of PACs	Type of interaction	Model	Effect	Reference
Polymers and oligomers of PACs from persimmon peel	Inhibition of α-amylase and α-glucosidase *in vitro*	PACs concentration: 5, 25, 50, and 100 µg/ml Substrate: starch and p-nitrophenyl α-D-glucopyranoside (pNPG)	Inhibition of α-amylase: 53.9% polymers; 4.6% oligomers (at 100 µg/ml) Inhibition of α-glucosidase: 74% polymers; 97.4% oligomers (at 100 µg/ml)	(105)

Water grape seed extract (WGSE, 26.7 mg PACs/g) and ethanol grape seed extract (EGSE, 32.6 mg PACs/g) of red grape seeds	Inhibition of α-amylase *in vitro*	Extract concentration: 400, 800, 1,300, and 1,800 ppm Substrate: starch	Inhibition of α-amylase: 74.86% EGSE; 52.48% WGSE (at 1,800 ppm) (enzyme preincubated with extract)	(106)

Four aqueous extracts from cinnamon bark (condensed tannins ranged between 0.12 and 0.15 g catechin equivalent/g extract)	Inhibition of α-amylase and α-glucosidase (maltase and sucrase) *in vitro*	Extract: Chinese, Ceylon, Saigon, and Thai cinnamon extracts (CEs) Substrate: starch, maltose, sucrose	Inhibitory activity [IC_50_ (mg/ml)] α-amylase: > Ceylon (1.23 mg/ml) < Saigon (>4 mg/ml) Maltase: > Thai (0.58 mg/ml) < Saigon (1.96 mg/ml) Sucrase: > Ceylon (0.42 mg/ml) < Saigon (>4 mg/ml)	(107)

Pycnogenol^®^ [65–75% procyanidins (PCs)]	Inhibition of α-glucosidase *in vitro*	Extract: pycnogenol^®^ compared with acarbose and green tea extract (both positive controls) Substrate: p-nitrophenyl α-D-glucopyranoside (pNPG)	Inhibitory activity [IC_50_ (μg/ml)] Pycnogenol^®^ (5.34 µg/ml) > green tea extract (19.74 µg/ml) > acarbose (1,010 µg/ml)	(108)

PACs from peanut skin extract (9 acetone fractions)	Inhibition of maltase and sucrase *in vitro*	Extract concentration: 1 mg/ml Substrate: maltose and sucrose	Inhibitory activity [IC_50_ (mg/ml)] Against maltase: epicatechin-(2β → O → 7,4β → 8)-[catechin-(6 → 4β)]-epicatechin (0.088 mg/ml) (trimeric PAC) Against sucrase: epicatechin-(4β → 8)-epicatechin-(2β → O → 7,4β → 8)-catechin (0.091 mg/ml) (trimeric PAC)	(110)

Condensed tannins from cranberry, grapes, and cocoa extracts	Inhibition of α-amylase and glucoamylase *in vitro*	Extract: different tannin:enzyme ratio Substrate: starch and maltose	Inhibitory activity (%): Against α-amylase (ratio 1:1): cocoa (14%); grape (28%); cranberry (55%) Against glucoamylase (ratio 0.01:1): cocoa, grape, and cranberry ~20% Against glucoamylase (ratio 1:1): cocoa (23%); grape (55%); cranberry (41%)	(103)

Octa-decyl silyl silica gel eluted fraction of polyphenols from peanut seed skin (39% PCs) and almond seed skin (30% PACs)	Inhibition of α-amylase and α-glucosidase *in vitro*	Substrate: starch, maltose, and sucrose	Peanut seed skin inhibitory activity (U/mg dry weight): α-amylase: 169; maltase: 4.41; sucrase: 4.69 Almond seed skin inhibitory activity [IC_50_ (μg/ml)]: α-amylase: 2.2; maltase: 468; sucrase: 627	(111, 112)

Cacao liquor PACs (CLPr)	Prevent elevation of blood glucose levels	Mice with diabetes mellitus and obesity (db/ob) feed with 0.5 and 1% of CLPr	↓ blood glucose levels at 4 and 5 weeks of age (1% CLPr) and 5 weeks of age (0.5% CLPr), compared with controls	(114)

PACs oligomers of persimmon leaf tea (PaW-PP)	Inhibition of α-amylase and maltase *in vitro* Blood glucose levels in rats	PaW-PP concentration: 240 µg/ml Substrate: starch and maltose Oral carbohydrate tolerance test: soluble potato starch (2.0 g/kg of body weight) and 1.0 ml of aqueous solution of PaW-PP (0, 100 or 300 mg/kg of body weight)	Inhibitory activity: 64% on α-amylase; 5.2% on maltase ↓ blood glucose levels at 30, 120, and 180 min after 100 mg/kg and in all time points after 300 mg/kg	(115)

Only one study has evaluated the effect of dietary PACs on lactase activity showing that tea epigallocatechin-3-gallate inhibits lactose hydrolysis by intestinal lactase *in vitro* (IC_50_ 74µM) at physiological luminal concentrations (113).

Although a great number of studies evaluated the effect of PACs on pancreatic amylase and disaccharidases, most of them were carried out *in vitro* and have inherent limitations due to the use of porcine or yeast enzymes, whose specificity may differ from these of human origin, and to the method employed for quantifying the enzymatic activity. For example, synthetic substrates are frequently used, that may be affected by the presence of PACs. In addition, they ignore the presence of other proteins normally present in the GI tract, like salivary proline-rich proteins, which may interfere with the inhibition of enzymes by PACs (102).

*In vivo* studies evaluating the interaction between PACs and the enzymes involved in carbohydrate digestion are scarce. Tomaru et al., for example, observed that dietary supplementation of diabetic obese mice with 0.5 or 1.0% cacao PACs [containing 2.49% catechin, 5.89% epicatechin, 3.93% PC B2, 2.38% PC C1, 3.17% cinnamtannin A2, and 0.48% galactopyranosyl-ent-(−)-epicatechin-(−)-epicatechin] dose dependently prevented the development of hyperglycemia (114). The administration of PAC oligomers from persimmon leaf tea to Wistar rats significantly decreased their blood glucose levels when compared with the placebo group (115).

#### Inhibition of Monosaccharide Transporters

The intestinal absorption of the monosaccharides resulting from starch and disaccharide digestion is mediated by transporters located in the brush-border membrane, on the apical side of the enterocytes. SGLT1 is an electrogenic transporter that depends on the Na^+^ gradient and mediates the absorption of glucose and galactose into the enterocyte (15, 102, 116). SGLT1 has a high affinity but a low transport capacity for glucose. GLUT5 is a facilitated transporter involved in fructose absorption (102, 116, 117), while GLUT2 is a facilitated transporter for glucose, galactose, and fructose; contrarily to SGLT1, it has low affinity but high transport capacity for glucose. GLUT2 was first located in the enterocyte basolateral membrane where it mediated the exit of the monosaccharides present in the cells to the systemic circulation during the postprandial period (118). More recently, the presence of GLUT2 has been described in intracellular vesicles. When the intraluminal concentration of glucose increases, i.e., after the intake of a meal rich in carbohydrates, GLUT2-containing vesicles translocate to the apical membrane where it contributes to glucose and fructose absorption. It has been proposed that two-third of the total amounts of glucose absorbed by the intestine in the postprandial period is through GLUT2. GLUT2 would be reinternalized in the cytoplasmic vesicles when the luminal concentration of glucose decreases or through insulin regulation (15, 102, 118–120).

The role of polyphenols in the regulation of the apical transporters has been widely studied (121–127). Several compounds have been shown to interfere with these transporters such as berry anthocyanins, apple polyphenols (phlorizin, quercetin, kaempferol, phloretin, and chlorogenic acid), helichrysum, and grapefruit (kaempferol-3-O-glucoside, chlorogenic acid-3-O-glucoside, naringenin-7-O-glucoside, naringenin diglycoside, kaempferol rutinoside, naringenin-7-O-rutinoside, and quercetin monoglucosides, among others) (121–126). Although the effect of PACs on these transporters was not evaluated so far, it is possible that they may exert certain activity, since flavanol monomers such as catechin, epicatechin, epigallocatechin, epicatechingallate, and epigallocatechingallate have been shown to inhibit SGLT1 or GLUT2 (128, 129). Kobayashi et al. observed that epicatechingallate and epigallocatechingallate (1mM) from tea reduced glucose uptake by rabbit brush-border membrane vesicles by 53 and 35%, respectively, whereas the inhibitory effects of catechin and epigallochatechin were not significant (128). In another study, all these flavanol monomers were shown to inhibit SGLT1-mediated glucose transport into Caco-2 cells (129).

### Effects on the Digestion and Absorption of Dietary Lipids

Triglycerides (TG) constitute the majority of the dietary lipids, while the contribution of cholesterol (CS) and phospholipids is much lower. Due to their hydrophobicity, lipids must be solubilized to be digested and posteriorly absorbed (130). Dietary fats are first emulsified in the stomach, a phenomenon that increases the rate of TG hydrolysis by the lipase and the release of diacylglycerol and free fatty acids (FFAs). Once in the small intestine, the fat emulsion is stabilized by bile salts, enabling the action of the colipase/pancreatic lipase (PL), CS ester hydrolase, and phospholipase A2 (PLA2) (130). PL hydrolyzes the TGs, releasing the fatty acids esterified in the carbon 1 and 3 of the molecule, and monoacylglycerol. Regarding CS ester hydrolase and PLA2, these enzymes hydrolyze CS esters and phospholipids, releasing free CS, FFAs, and lysolecithin (130). As these final products of digestion (monoglycerides, FFAs, CS, and lysolecithin) are hydrophobic, they must be incorporated into biliary mixed micelles as they are released in the lumen. This process of solubilization allows them to diffuse across the unstirred water layer until the enterocyte surface, where they are released from the micelles. Posteriorly, they enter into the absorptive cells by passive diffusion according their concentration gradient or by using specific transporters such as FAT/CD36, FATP4, and FABPpm for FFAs and Niemann–Pick C1-like protein 1 (NPC1L1) for CS (131, 132). In the enterocytes, FFAs are reesterified with glycerol or CS, and the resulting TG and CS ester are subsequently incorporated to chylomicrons and exported into lymphatic circulation, to finally end up in the bloodstream. Bile salts are reabsorbed in the terminal ileum through the apical sodium bile acid transporter, they reach the circulation and are taken up by the liver and resecreted by the biliary system. This enterohepatic circulation takes place 10 times per day so that less than 5% of bile acids enter the large intestine during this period for fecal elimination (133).

Similar to carbohydrates, the enzymes and transporters involved in lipid digestion and absorption are subjected to the action of dietary polyphenols. Most of the studies have focused on the inhibitory activity on of these compounds on PL, PLA2, or bile salts, due to their implication in fat absorption and their potential to prevent obesity and its complications. Fewer studies have focused on their interaction with CS esterase or lipid transporters (Table 4).

**Table 4:** **Impact of proanthocyanidins on enzymes and transporters involved in the digestion and absorption of lipids**.

Type of PACs	Type of interaction	Model	Effect	Reference
Water grape seed extract (WGSE, 26.7 mg PACs/g) and ethanol grape seed extract (EGSE, 32.6 mg PACs/g) of red grape seeds	Inhibition of pancreatic lipase (PL) *in vitro*	Extract concentration: 400, 800, 1,300, and 1,800 ppm Substrate: olive oil emulsion	Inhibition of lipase: 52.66% EGSE; 45.44% WGSE (at 1,800 ppm) (enzyme preincubated with extract) 61.41% EGSE; 42.63% WGSE (at 1,800 ppm) (substrate preincubated with extract)	(106)

Apple polyphenol extract (AP) and the procyanidins (PCs) contained in the extract	Inhibition of PL *in vitro*	Substrate: 4MUO	Inhibition of lipase [IC_50_ (μg/ml)]: PCs (1.4 µg/ml) > AP (5.6 µg/ml) > other polyphenol fraction (115.9 µg/ml)	(135)
		
	Triglyceride (TG) absorption in mice	1,000 mg/kg of AP compared with control (water) and 10 ml/kg body weight of corn oil	AP completely prevent the increase of plasma TG level	
		
	TG absorption in humans	600 mg of AP and 40 g of TG	Inhibition of TG elevation at 6 h after ingestion	

Cocoa (PCs) (>85% purity) [degree of polymerization (DP) between 2 and 10]	Inhibition of PL and secreted phospholipase A2 (PLA2) *in vitro*	PL: PCs: 0–20µM and 4-NPB as substrate. Orlistat as control PLA2: PCs: 0–100μM and Red/Green BODIPY PC-A2, 1.67µM as substrate	PCs with DP ≥ 5 inhibited PL by 37 to 53% at 20µM. Orlistat 72% at 10µM PCs with DP between 2 and 5 inhibited PLA2 by 46–74% at 100µM PCs with DP between 6 and 10 inhibited PLA2 by approximately 90% at 50µM (IC_50_ < 5µM)	(136)

Tannins from persimmon	Capacity to bind primary and secondary bile acids *in vitro* and *in vivo*	Tannins concentration: 1% (w/v). Bile acids concentration: 1 mM solutions. Cholestyramine positive control	Tannins adsorbed approximately 80% of all bile acids (cholic, taurocholic, glycocholic, and deoxycholic acid), similar to a cholestyramine	(138)
			
		Mice supplemented for 14 days with a tannins diet [1% (w/w)] Cholestyramine as positive control	Tannin supplementation: twofold more excretion of bile acids in feces compared with a control (without supplementation)	

Grape seed extract (GSE, 49.8% PCs)	Inhibition of PL	GSE 4.75–0.62 mg/ml. Orlistat as positive control	Inhibitory activity: GSE (IC_50_ 44.5 mg/ml); Orlistat (IC_50_ 3.7 mg/ml)	(139)
		
	Inhibition of pancreatic cholesterol (CS) esterase	GSE 50–3.12 µg/ml. Simasvatin as positive control	Inhibitory activity: GSE (IC_50_ 27.27 µg/ml); Simasvatin (IC_50_ 0.08 µg/ml)	
		
	Effect on CS micellization	GSE at 10, 20, and 40 mg/ml on artificially micelles. Gallic acid as positive control	Inhibition of CS solubility: gallic acid (27.26%) > GSE 40 mg/ml (11.87%) > GSE 20 mg/ml (6.84%) > GSE 10 mg/ml (3.18%)	
		
	Capacity to bind bile acid	GSE 1 mg/ml. Bile acid 2 mM. Cholestyramine as positive control	% Bile acid binding: glycodeoxycholic (70%) > taurocholic (25%), both similar to cholestyramine. Taurodeoxycholic acid was slightly bound	
		
	Serum TG and CS concentrations	Oral administration of 5 ml/kg body weight of olive oil emulsion (3.33 ml of olive oil, 44.3 mg of cholic acid, 0.48 g of CS, and 1.67 ml of distilled water) GSE: 100, 250, and 500 mg/kg	↓ in serum TG concentrations (2–6 h after administration) at 250 and 500 mg/kg GSE [area under the curve (AUC) 19 and 27% lower than control group, respectively] Suppression of the increase in serum CS concentration (after 4 h of loading fat emulsion) at 250 and 500 mg/kg (AUC 8 and 11% lower than in control group, respectively)	

Aqueous CE (4.1% type-A polymers)	Expression of genes of Niemann–Pick C1-like protein 1 (NPC1L1) and CD36	Enterocytes treated with 10 or 100 µg/ml of CE for 0, 0.5, 2, and 4 h	↓ NPC1L1 mRNA at 2 h (10 µg/ml) and at 4 h (100 µg/ml) ↓ CD36 mRNA at 4 h (10 µg/ml) and at 0.5 h (100 µg/ml)	(140)

Grape seed proanthocyanidin extract (GSPE)	Intestinal expression of NPC1L1 and CD36	Rats fed with lard (2,5 ml/kg of body weight), supplemented or not with GSPE (250 mg/kg of body weight)	Lard induces a decrease in the expression of all genes evaluated. GSPE do not induce changes in the expression of genes	(141)

Polyphenol extracts from grape (41% PACs), cranberry (32% PACs), avocado (29% PACs), and apple (22% PACs)	Inhibitory activity on PL *in vitro*	Lipase: 2 mg/ml. PACs extracts: 0; 0.065; 0.125; 0.25; 0.5; and 1 mg/ml. Substrate: 4-MUO (4-metilumbeliferil oleate) (0.1mM)	Inhibitory activity: Grape > cranberry > avocado > apple	(137)
		
	Plasma TG concentrations *in vivo*	Subjects: normal weight (NW) and overweight/obese (OW/Ob) Extract: 1 g of extract or placebo High-fat test meal: 63 g fat	Absorption of TG was 2 times higher in the OW/Ob than in the NW subjects and was unaffected by the treatment

#### Inhibition of Enzymes Involved in Lipid Digestion

Hassan reported that an ethanol extract from grape seed was more efficient than a water extract in inhibiting TG hydrolysis by PL *in vitro* when they were preincubated with the enzyme (52.7 vs 45.4%, respectively) or with its substrate (61.4 vs 42.6%, respectively). Such effect was related to the PACs content of the extracts (106). An apple polyphenol extract containing 65.7% PCs and 12.5% flavan-3-ols almost completely inhibited PL activity in a dose-dependent manner (134). The purified PC fraction showed the higher inhibitory activity, compared to the other polyphenols fractions, and the effect was mainly associated with the DP [DP ≥ 5 had the highest inhibitory activity, especially the heptamer fraction (IC_50_ = 0.7 μg/ml)]. Results in mice indicate that 1,000 mg/kg of the apple extract fully prevented the increase of plasma TG after the administration of 10 ml corn oil/kg body weight, compared to the control group. In humans, the elevation of postprandial plasma TG was significantly inhibited after the intake of 600 mg apple extract together with 40 g dietary fat (135). In another study, the effect of purified cocoa PCs (>85%; DP between 2 and 10, B-type) against PL and PLA2 was determined *in vitro*. PCs with DP ≥ 5 inhibited PL, while those with a DP between 2 and 5 inhibited PLA2, and those with DP between 6 and 10 inhibited PLA2 by approximately 90% at 50µM (136). The authors conclude that cocoa PCs has higher inhibitory activity against PLA2 than against PL and suggest that DP is an important factor in determining the potency of these compounds. We recently compared the lipase inhibitory effect of PAC-containing polyphenol extracts from grape, cranberry, avocado, and apple (137). The most to least efficient extracts were grape > cranberry > avocado > apple. The strongest lipase inhibitory activity was exerted by the extract with the higher PAC content and higher DP (9.8) and the only ones containing galloyl moieties. Accordingly, we observed that the PAC content of the extracts correlated (*r* = 0.85; *p* < 0.001) with their lipase inhibitory activity, and a similar correlation was also observed when considering the PAC DP. However, when 1 g of the grape extract was administered to normal-weight or overweight/obese subjects simultaneously with a high-fat breakfast, the postprandial increase of plasma TG was not affected compared to the placebo (137). It is important to consider that in humans, PL is released in excess into the intestinal lumen and that, independently of the inhibitory activity exhibited by the extract *in vitro* against this enzyme, the amounts of PACs ingested may be insufficient to inhibit the enzymatic activity completely.

Another interesting target for PACs to inhibit lipid absorption is bile salts. Matsumoto et al. (138) investigated the ability of persimmon tannins to bind primary and secondary bile acids *in vitro* and *in vivo*. These PACs had a high DP and were composed by epicatechin, epigallocatechin, epicatechin-3-O-gallate, and epigallocatechin-3-O-gallate. At a concentration of 1% (w/v), PACs adsorbed approximately 80% of the primary and secondary bile acids *in vitro*, similar to cholestyramine. In mice fed a diet supplemented with 1% persimmon tannins for 14 days, a twofold increase of fecal bile salt excretion was observed, compared to the control, not supplemented, group. However, this increase remained lower than that observed in the animals treated with cholestyramine. Such interference with bile salts might affect the stabilization of fat emulsion in the intestinal lumen, and/or the formation of biliary micelles.

A grape seed extract (49.8% PCs) was shown to inhibit PL and CS esterase activities, but less than their respective positive controls, orlistat and simvastatin. To study the effect of the extract on CS micellization, the authors evaluated the solubility of CS in artificially prepared micelles in presence of different concentration of the extract. They observed a decrease of CS solubility, but less than with the positive control gallic acid (139). With respect to bile acid binding capacity, the extract binds strongly glycoldeoxycholic and taurocholic acids (70 and 25%, respectively) at a concentration of 1 mg/ml, similar to the effect of cholestyramine at the same concentration. Rats fed a high-fat emulsion with 250 or 500 mg/kg extract showed a significant diminution in the postprandial plasma TG concentrations between 2 and 6 h, as reflected by changes in the area under the curve (AUC_TG_) (19 and 27% lower than the control group). At these same doses, the extract significantly suppressed the postprandial increase in serum CS concentrations (AUC 11% lower than the control group). It is therefore possible that this extract can be used as therapeutic strategy to prevent hyperlipidemia and obesity due to its capacity to improve plasma lipid profile.

#### Inhibition of Lipid Transporters

A number of studies have focused on the interactions between polyphenols and intestinal lipids transporters. An aqueous CE containing PACs (4.1% type-A polymers) has been shown to decrease the mRNA levels of CD36 (a FFA transporter) and NPC1L1 (implicated in the intestinal uptake of CS) in small intestine enterocytes (140). In opposition to these results, Quesada et al. did not report any effect of a grape seed extract on the expression of these transporters in rats fed a diet with lard (141).

#### Inhibition of Lipopolysaccharide (LPS) Absorption

In our previously described study (137), we also address the capacity of PACs to bind bacterial LPS, as previously by described by Delehanty et al. (142). In fact, it has been proposed that LPS from intestinal Gram-negative bacteria could enter the enterocytes and be incorporated into the chylomicrons to be excreted to the lymphatic system and bloodstream. Accordingly, the presence of dietary lipids in the intestinal lumen would stimulate LPS absorption and, eventually the development of metabolic endotoxemia (143, 144). We confirm that the grape extract bound LPS *in vitro*, inhibiting its union to polymyxin B and that, when administered to the volunteers after the high-fat meal, it significantly decreased the elevation of postprandial plasma LPS associated with that of TG in the volunteers (137). This phenomenon constitutes a new mechanism by which PACs may decrease systemic inflammation.

In conclusion, some evidence exist that dietary PACs interfere with the different events involved in intraluminal lipid processing, including enzymatic hydrolysis, micellization, and uptake of lipid digestion products by the intestinal epithelial cells. However, most of the studies have focused on the interaction between PACs and PL *in vitro* and, in some cases, in animal models, which do not accurately represent the situation occurring in the organism. Considering these points, further studies are necessary to elucidate the role of PACs in intestinal fat digestion and absorption in humans.

### Effects on the Digestion and Absorption of Dietary Proteins

The digestion of dietary protein occurs first in the gastric and intestinal lumen through the action of HCl/pepsin and pancreatic proteases, including pepsin, trypsin, chymotrypsin, elastase, carboxypeptidase A, B, and aminopeptidase, and concludes with peptidases located in the brush-border membrane and cytoplasm of the enterocytes (130, 145). Pepsin and pancreatic proteases hydrolyze proteins in amino acids, di-, tri-, and oligopeptides. Posteriorly, amino acids are absorbed into the enterocytes through specific transporters according to their chemical structure (146), while di- and tripeptides are transported by the proton-dependent cotransporter, Pept-1 (130, 147). Oligopeptides need further hydrolysis by brush-border proteases to yield absorbable molecules (130). In the absorptive cells, the di- and tripeptides are hydrolyzed to amino acids by cytosolic peptidases, and these are exported to the bloodstream by facilitated diffusion (130).

#### Inhibition of Enzymes and Transporters Involved in Protein Digestion and Absorption

As previously stated, PACs display a high affinity for proteins, particularly for these with high-proline content. Accordingly, they may affect the bioavailability of dietary proteins by decreasing their digestibility, either directly by binding them or indirectly by inhibiting enzymatic activities (Table 5). These processes contribute to the fact that condensed tannins are sometime considered as antinutritional factors.

**Table 5:** **Impact of proanthocyanidins on enzymes and transporters involved in the digestion of proteins**.

Type of PACs	Type of interaction	Model	Effect	Reference
Oligomeric procyanidins (OPC) of grape seed	Inhibition of trypsin *in vitro*	Trypsin: 0.072 g/l Substrate: BApNA	OPC at 313 mg/l had the highest inhibitory activity. At 233 mg/l fractions 4 and 5 had the higher inhibitory activity	(148)

Condensed tannins from 4 fodder plants (PACs from oligomer to octamer)	Inhibition of trypsin *in vitro* and *in vivo*	Substrate: BApNA Rats feed with tannin containing diet (10 g/kg)	Positive correlation between degree of polymerization and inhibitory activity on trypsin (*r* = 0.928) and protein precipitation capacity (*r* = 0.855) ↓ in activity of trypsin in the upper, middle, and lower segments of intestine	(150)

Procyanidins from grape seed	Inhibition of pancreatic elastase *in vitro*	Pancreatic elastase (PPE) (0.8 µM), Suc-(Ala)3-p-nitroanilide (250 µM) as substrate	Inhibitory activity: Oligomeric fraction: IC_50_ 16 µM Tetramer fraction: IC_50_ 585.1 µM Trimer C2: IC_50_ 5,863.3 µM	(151)

A maximal inhibitory activity of trypsin *in vitro* was reported for grape seed PACs at a concentration of 313 mg/l; the enzymatic inhibition correlated with PC DP (148). In another study, the same authors investigated the mechanism by which trypsin is inhibited by PC dimer B3 (149). They reported that, at low concentration, specific interactions mediated by hydrogen bonds occur between the hydroxyl groups of the dimer and the amide and carbonyl group of the protein backbone, while at high concentration, those interactions were not specific. Horigome et al. also reported a positive correlation between the PAC DP of four fodder plants and their capacity to precipitate proteins and inhibit trypsin activity (150). In addition, they observed that the preincubation of bovine serum albumin with PACs inhibited its digestion. Rats fed a tannin supplemented diet (10 g/kg) displayed a significantly reduced trypsin activity in their intestine. When antibiotic treated rats (to eliminate the effect of bacterial enzymes) were fed with a tannin supplemented diet (20 g/kg), they observed a significant decrease in nutrient digestibility. Based on these results the authors conclude that the formation of insoluble enzyme–tannin complexes is responsible of the inhibition of trypsin activity. Brás et al. studied the effect of dimer B3, trimer C2, tetramer, and oligomer fractions of PCs on pancreatic elastase activity. The inhibitory activity increased with the PCs molecular weight, being the oligomer fraction the most potent inhibitor (approximately 90%; IC_50_ = 16μM) (151). In another study, these authors reported that elastase, in the presence of PCs, undergoes slight changes in its secondary (R-helix to β-sheet) and tertiary structures, and forms insoluble aggregates (152).

Fewer studies have addressed the influence of PACs on the others enzymes involved in protein digestion. Uchida et al. showed that the inhibitory activity of condensed tannins from Rhei Rhizoma against the angiotensin converting enzyme increased with the PACs DP (153). The PC B-5 3,3′-di-*O*-gallate had the highest angiotensin converting enzyme inhibitory activity, and the authors indicate that this activity was protein-specific as this PACs did not inhibit other enzyme activities such as trypsin, chymotrypsin, leucine aminopeptidase, carboxypeptidase A, and urinary kallikrein. No studies were found regarding the interaction between PACs and intestinal amino acids and peptides transporters.

The fate of the PAC-bound proteins in the intestinal and colonic lumen remains unclear. An increased flux of indigested proteins in the colon may result in a major production of toxic metabolites derived from their fermentation by the intestinal microbiota (IM), with negative consequences to the host (154). However, PACs are used in ruminant nutrition to reduce protein degradation in the rumen (155), and it is possible that this phenomenon also occurs in the human colon. PAC-bound proteins, therefore, should be eliminated in stools.

## Effects of PACs in Diarrhea and GI Motility

Cássia Santos et al. evaluated the effect of a methanolic extract from leaves of *B. intermedia* containing catechin derivatives and oligomeric PACs in a rodent model of castor oil-induced diarrhea. The extract was shown to prevent or revert the diarrhea, decreasing fluid accumulation and the emission of watery stools, probably through the stimulation of intestinal opioid receptors, without affecting intestinal motility (156). In a similar study, a fraction from *Chiranthodendron pentadactylon* flowers rich in flavan-3-ols was evaluated using rat jejunal loops exposed to cholera toxin (3 µg). (−)-Epicatechin showed the best antisecretory activity (ID_50_ = 8.3 μm/kg), like that of the antisecretory drug loperamide (ID_50_ = 6.1 µm/kg), and better than (+)-catechin (ID_50_ = 51.7 μm/kg) and other compounds presents in the extract (flavonol glycosides). Such observations support the traditional use of *C. pentadactylon* flowers in the treatment of dysentery in the Mexican traditional pharmacopeia (157). In another study, the effect of *Aronia melanocarpa* fruit juice (rich in condensed tannins) was shown to significantly reduce the intestinal transit in charcoal-administered rats (158). Interestingly, a patent (No US 7,341,744 B1) “Method of treating secretory diarrhea with enteric formulations of proanthocyanidins polymer” was recently registered by Rozhon et al. that describes the pharmaceutical formulation of a proanthocyanidin polymer isolated from *Croton* spp. or *Calophyllum* spp., useful for the treatment and prevention of secretory diarrhea (159).

## The PACs in the Colon

The human colon harbors a highly complex microbial ecosystem that includes bacteria, yeasts, fungi, virus, and phages. More than 1,000 species of bacteria have been described, most of them anaerobic, and with counts reaching 10^11^–10^12^/g of intracolonic content (160–166). These microorganisms express a great number of enzymes capable of metabolizing the majority of the substrates reaching the colon, including xenobiotics and PACs (167). The gut microbiota exhibits metabolic, nutritional, and protective functions important for the host health (161, 165). It is involved in energy salvage from the dietary compounds non-digested and absorbed in the small intestine, vitamin synthesis, and in the metabolism of bile salts and xenobiotics. The IM also exerts a protective function, decreasing the risk of pathogen overgrowth in the colonic lumen, stimulating the local immune system and contributing to the development of oral immune tolerance (154, 161, 163). The dominant bacterial phyla constituting the IM are Firmicutes and Bacteroidetes and, in lower proportions, Proteobacteria, Actinobacteria, and Verrucomicrobia (164, 165), while the main bacterial genera are *Clostridium, Bacteroides, Prevotella, Eubacterium, Ruminococcus, Fusobacterium, Peptococcus*, and *Bifidobacterium* (160). Some genera are more particularly considered as beneficial for the host like *Bifidobacterium, Lactobacillus, Faecalibacterium prausnitzii* (a butyrate-producing bacteria), and *Akkermansia muciniphila*, while others are considered as potentially harmful like *Staphylococcus*, some species of the *Clostridium* genus (*C. perfringens, C. difficile*) and *Pseudomonas*, which have been associated with diarrhea, systemic infections, liver damage, cancer, and encephalopathy (162). Among the numerous metabolites produced by the IM, the most widely studied are those produced by the fermentation of the dietary fiber in the colon, i.e., the short chain fatty acids (SCFAs) acetate, propionate, and butyrate (161). The decrease of colonic luminal pH induced by SCFAs reduces the risk of pathogen overgrowth and improves mineral solubility and absorption (163). They also act, promoting the integrity of the gut barrier function and host satiety (168). Butyrate is also considered as a preferential substrate for the colonocytes; in addition, it exerts antiinflammatory and antitumoral activities, this latter by favoring the differentiation and apoptosis of epithelial cells. Regarding acetate and propionate, they have been implicated in the regulation of lipid metabolism in the liver (161, 163). However, some of the metabolites produced by the colonic fermentation of amino acids including ammonia, hydrogen sulfide, indol, and phenol compounds, among others, can exert deleterious effects on the colonic mucosa and host health. Indeed, these metabolites affect colonocyte oxidative metabolism and cellular respiration, produce genomic DNA damage, affect the integrity of the barrier function, and also act like pro-carcinogens and promoters of colorectal cancer as well as inflammatory bowel diseases (IBD) (154, 165, 169).

The composition of the IM is influenced by many factors such as the age, gender, and genetic background of the host, the consumption of xenobiotics, antibiotic, and other drugs, the existence of physical or psychological stress, environmental and dietary factors, this latter being probably the most important (160, 161, 164). Accordingly, probiotics and prebiotics have been typically used as a strategy for the nutritional management of the IM composition and its metabolic/immunological activities (165, 170).

### Effect on the IM

As described above, a great proportion of the dietary polyphenols, including PACs, remain undigested in the intestine and reach the colon, where they are used as substrates by specific bacterial populations, stimulating their growth much like prebiotics do. In addition, PACs with high DP exert bacteriostatic and eventually bactericide effects that might also contribute to the modulation of IM composition and bacterial adhesion to colonocytes (164, 171).

Several studies have investigated the effect of PAC intake on the composition of the IM. In an *in vitro* model of colonic fermentation, the addition of (+) catechin (150 mg/l) for 48 h increased significantly the growth of *C. coccoides*–*E. rectale* group, *Bifidobacterium* spp., and *E. coli* and inhibited that of *C. histolyticum*. In the same conditions, the incubation with epicatechin only increased the growth of *C. coccoides*–*E. rectale* group (172). In a similar model, a grape seed extract with 28% PACs (600 mg/l) promoted the growth of *Lactobacillus/Enterococcus* group at 5 and 10 h, while the extract with 78% PAC at the same concentration only decreased *C. histolyticum* (173). In a recent interventional study, healthy volunteers had to ingest a dairy-based cocoa beverage with high (494 mg) or low (29 mg) flavanol content once a day for 4 weeks (174). The high flavanol beverage was composed of 110 mg monomers (catechin and epicatechin), 99 mg dimers, and 285 mg polymers (trimers to decamers), while the low flavanol beverage only contained 6, 11, and 12 mg of these compounds, respectively. The high flavanol drink significantly increased the counts of *Bididobacterium, Lactobacillus*, and *Enterococcus* and decreased those of *C. histolyticum*, while the low flavanol drink increased *C. histolyticum*. Both beverages stimulated the growth of the *E. rectale/C. coccoides* group. Accordingly, cocoa flavanols display a prebiotic potential by promoting a healthy IM in humans. In another human study, the effect of 2-week administration of 0.19 g/day of a PAC-rich grape seed extract (DP 2–15) was evaluated on the IM composition and fecal odor in healthy adults (175). An increase of *Bifidobacterium* and a non-significant decrease of *Enterobacteriaceae* were reported. Interestingly, the concentrations of potentially harmful bacterial metabolites such as ammonia, phenol, *p*-cresol, 4-ethylphenol, indol, and skatol (eventually implicated in the development of colorectal cancer) tended to decrease. Methyl mercaptan gas concentrations and fecal odor decreased significantly. Therefore, in this study, the modification of the IM by PACs was associated with a healthier environment in the colonic ecosystem.

The study of PAC effects on the IM is not restricted to humans. In ruminant nutrition, PACs are recognized to modulate fermentation processes in the rumen, inhibiting methane-producing archaea, decreasing bloating and protein degradation, and favoring the formation of conjugated linoleic acid. Some studies suggest that body weight gain, milk yields, and reproductive performance could be also improved by incorporating tannins in the animal diet, even if there is not yet a clear explanation for these beneficial effects (155).

### Transformation of PACs by Gut Microbiota

The enzymatic degradation of flavonoids by the colonic microbiota results in a huge array of new metabolites. Bacterial enzymes may catalyze many reactions including hydrolysis, dehydroxylation, demethylation, decarboxylation, and deconjugation (176). Many flavonoids undergo ring-fission in which their C-ring is degraded, A-ring forms hydroxylated aromatic compounds, and B-ring phenolic acids derivatives (177). *Clostridium* and *Eubacterium* have been proposed as the main bacterial genera involved in the metabolism of phenolic compounds including flavan-3-ols (178). It is considered that about 40% of the flavan-3-ols ingested with green tea are converted to phenolic acid metabolites in the colon, which are excreted in urine. About 8% of them are methyl, glucuronide, and sulfate derivatives of flavanols, which reflect the fact that these metabolites were previously absorbed by the colonic epithelium (179).

The first study carried out in humans by Das in 1971 identified 11 metabolites in urine after (+)-catechin intake; among the most important were m-hydroxyphenylpropionic acid, δ-(3,4-dihydroxyphenyl)-γ-valerolactone, and δ-(3-hydroxyphenyl)-γ-valerolactone (180). These results were confirmed more recently by Li et al. who described phenyl-valerolactones as the main tea catechin metabolites produced by gut microorganisms and detected in human urine and blood (181). Consistently, Tzounis et al. reported that the incubation of (−)-epicatechin or (+)-catechin with fecal bacteria led to the generation of 5-(3′,4′-dihydroxyphenyl)-gamma-valerolactone, 5-phenyl-gamma-valerolactone, and phenylpropionic acid (172). Valerolactones were also detected in human urine by Ottaviani et al. after the consumption of flavanols and PCs (182). Aura et al. observed that 3-hydroxyphenyl propionic acid and 3-phenylpropionic acid were the main metabolites originated from (+)-catechin and (−)-epicatechin by human microbiota (183). These results are consistent with those described by Gonthier et al. who also identified urinary and plasma 3-hydroxybenzoic acid and 3-hydroxyhippuric acid in rats fed a diet supplemented with red wine polyphenols (184). Flavanol degradation by the microbiota seems to be a rapid process completed in 4–8 h, as reported in a “pig cecum *in vitro* model” (185). In another study with pig microbiota, it was shown that about 80% of PC A2 and 40% of cinnamtannin B1 were degraded after 8 h of incubation (186). 3-O-Gallate derivatives of epicatechin and epigallocatechin were extensively metabolized by a human fecal microbiota after 24-h-incubation but remained unaffected in presence of rat fecal microbiota, even after 48-h-incubation. These results suggest differences in the microbiota and their associated metabolic ability between both species (187). Phenylvaleric, phenylpropionic, cinnamic, phenylacetic, and benzoic acids as well as conjugated derivatives of benzoic acid have been identified in urine samples of rats fed with PC dimer B3, trimer C2, and catechin polymer (74). In another study, the main metabolites identified after the *in vitro* fermentation of purified PAC dimers with a human fecal microbiota were 2-(3,4-dihydroxyphenyl) acetic acid and 5-(3,4-dihydroxyphenyl)-γ-valerolactone (167). *m*-hydroxyphenylpropionic acid, ferulic acid, 3,4-dihydroxyphenylacetic acid, *m-*hydroxyphenylacetic acid, vanillic acid, and *m*-hydroxybenzoic acid were identified in urine samples of human volunteers after cocoa intake (188). These results were confirmed by Urpi-Sarda et al. who reported increased urinary concentrations of caffeic acid, ferulic acid, 3-hydroxyphenylacetic acid, vanillic acid, 3-hydroxybenzoic acid, 4-hydroxyhippuric acid, and hippuric acid in humans, while in rats were identified 3,4-dihydroxyphenylpropionic acid, *m*-coumaric acid, 3-hydroxyphenylacetic acid, protocatechuic acid and vanillic acid (189). These slight differences in the metabolite profile probably reflect interspecies differences in their microbiota composition. Ward et al. detected urinary 3-hydroxyphenylpropionic acid and 4-O-methylgallic acid after regular consumption of PAC-containing grape seed extract in humans (190). Interestingly, Jenner et al. measured the concentrations of dietary polyphenols and their bacterial metabolites in fecal waters from healthy omnivorous subjects under normal diet (i.e., without additional supplementation with fruits and vegetables). The major components detected were phenylacetic acid (479µM), 3-phenylpropionic acid (166µM), 3-(4-hydroxy)-phenylpropionic acid (68µM), 3,4-dihydroxycinnamic acid (52µM), benzoic acid (51µM), 3-hydroxyphenylacetic acid (46µM), and 4–hydroxyphenylacetic acid (19µM). Other phenolic acids ranged between 0.04 and 7µM in fecal waters (19). The bacterial degradation of black tea polyphenol-rich and red wine/grape juice extracts by the colonic microbiota were compared in an *in vitro* five-stage GI model (SHIME^®^). The levels of gallic acid and 4-hydroxyphenylpropionic acid remained elevated throughout the colon with red wine/grape juice feeding, while these compounds were consumed in the distal colon and 3-phenylpropionic acid was strongly produced during a polyphenol-rich black tea extract feeding. The gut microbial production of phenolics was dependent on their location in the colon and the extract source (191).

The PAC DP is an important factor affecting the microbial metabolism of these compounds. The presence of PACs with high DP prevents the *in vitro* microbial metabolism of PACs (192), and A-type PACs are also more resistant to microbial degradation than B-type PACs (193). The main flavanol bacterial metabolites described in these studies are summarized in Table 6.

**Table 6:** **Flavanol metabolites identified from microbial conversion *(in vitro)* or in body fluids *(in vivo)***.

Flavanols	Metabolites		Reference
*(*+)*-catechin*	3-hydroxyphenylpropionic acid	Human urine and feces	(180)
	
	5-(3′,4′,5′-trihydroxyphenyl)-γ-valerolactone 5-(3′,4′-dihydroxyphenyl)-γ-valerolactone	Human urine and blood	(181)
	
	3-hydroxyphenylpropionic acid 3-hydroxybenzoic acid 3-hydroxyhippuric acid 3,4-dihydroxyphenylpropionic acid 3,4-dihydroxyphenylacetic acid Ferulic acid	Rat urine and plasma	(184)
	
	3,4-dihydroxyphenylpropionic acid (inoculum A) 3-hydroxyphenylpropionic acid (inoculum A) 3-phenylpropionic acid (inoculum A) 3,4-dihydroxyphenylvaleric acid (inoculum B) 3-hydroxyphenylvaleric acid (inoculum B)	Human fecal microbiota	(183)
	
	5-(3′,4′-dihydroxyphenyl)-γ-valerolactone 5-phenyl-γ-valerolactone Phenylpropionic acid	Human fecal microbiota	(172)
	
	3,4-dihydroxyphenylpropionic acid 4-hydroxyphenylacetic acid 4-hydroxybenzoic acid	Porcine fecal microbiota	(185)

*(*−*)-epicatechin*	3-hydroxyphenylpropionic acid	Human urine and feces	(180)
	
	3-hydroxyphenylpropionic acid (inoculum A) 3-phenylpropionic acid (inoculum A) 3,4-dihydroxyphenylvaleric acid (inoculum B) 3-hydroxyphenylvaleric acid (inoculum B)	Human fecal microbiota	(177)
	
	5-(3′,4′-dihydroxyphenyl)-γ-valerolactone 5-phenyl-γ-valerolactone Phenylpropionic acid	Human fecal microbiota	(172)
	
	3,4-dihydroxyphenylpropionic acid 4-hydroxyphenylacetic acid 4-hydroxybenzoic acid	Porcine fecal microbiota	(185)

*(*−*)-epigallocatechin and (*−*)-epigallocatechingallate*	4-phenylacetic acid 3- and 4-hydroxybenzoic acid Gallic acid	Porcine fecal microbiota	(185)

*Proanthocyanidins*	3-hydroxyphenylpropionic acid 3,4-dihydroxyphenylacetic acid 3-hydroxyphenylvaleric acid Phenylacetic acid 3-hydroxyphenylacetic acid 3,4-dihydroxyphenylacetic acid 3-hydroxybenzoic acid 4-hydroxybenzoic acid Hippuric acid Vanillic acid Caffeic acid Ferulic acid 3-Methoxy, 4-hydroxyphenilvalerolactone 3,4-dihydroxyphenylvalerolactone	Rat urine	(74)
	
	3-hydroxyphenylpropionic acid Ferulic acid 3,4-dihydroxyphenylacetic acid 3-hydroxyphenylacetic acid Vanillic acid 3-hydroxybenzoic acid 4-hydroxybenzoic acid	Human urine	(188)
	
	Caffeic acid Ferulic acid 3-hydroxyphenylacetic acid Vanillic acid 3-hydroxybenzoic acid 4-hydroxyhippuric acid 3,4-dihydroxyphenylpropionic acid	Human and rat urine	(189)
	
	3-hydroxyphenylpropionic acid 4-O-methylgallyc acid	Human urine	(190)
	
	2-(4-hydroxyphenyl)acetic acid 2-(3-hydroxyphenyl)acetic acid 3-(4-hydroxyphenyl)propionic acid 3-hydroxyphenylpropionic acid 5-(3-hydroxyphenyl)valeric acid 3-phenylpropionic acid	Human fecal microbiota	(18)
	
	2-(3,4-dihydroxyphenyl)acetic acid 5-(3,4-dihydroxyphenyl)-γ-valerolactone	Human fecal microbiota	(167)
	
	3-(4-hydroxyphenyl)propionic acid 3-hydroxyphenylpropionic acid 3,4-dihydroxyphenylacetic acid 4-hydroxyphenylacetic acid	Porcine fecal microbiota	(186)

Whether these PAC metabolites can be used by the cells as a source of energy is unclear. A recent study showed that 3,4-dihydroxyphenylacetic acid, a microbial metabolite of quercetin and PACs, can protect against the mitochondrial dysfunction induced by CS in Min6 pancreatic β-cells (194). CS (320µM) decreased the mitochondrial membrane potential, the intracellular concentrations of ATP and the rate of oxygen consumption, while 3,4-dihydroxyphenylacetic acid (100–250µM) prevented, in a concentration-dependent manner, these mitochondrial function alterations. At 250µM, this metabolite was capable of preventing the drop in oxygen consumption and complex I activity, suggesting that 3,4-dihydroxyphenylacetic acid improves the energetic metabolism of these cells.

### Protective Effect of PACs in Colonic Inflammation

Inflammatory bowel diseases, mainly Crohn’s disease and ulcerative colitis, are considered as a growing problem of public health in the world. The etiology of these diseases remains poorly understood. Although the mechanisms underlying the occurrence of Crohn’s disease and ulcerative colitis differ, both diseases are characterized by a chronic inflammation and increased oxidative stress in the mucosa, with an inappropriate activation of the immune system (195). Considering that dietary PACs exhibit antiinflammatory, immunomodulatory, and antioxidant properties, a number of studies have tested their impact in animal models of IBD (Table 7).

**Table 7:** **Protective effect of proanthocyanidins in animal models of colonic inflammation**.

Extract	Extract administration	Experimentally induced colitis	Effect	Reference
Apple PACs	0.1, 0.3, or 1% in drinking water *ad libitum* for 14 days before DSS treatment	DSS in drinking water *ad libitum*	↓ colonic damage ↓ mortality rate ↓ body weight loss ↑ TCRγδ and TCRαβ T cells in IEL	(196)
	
	1% in drinking water *ad libitum* for 7 days before oxazolone administration	7.5 mg/ml oxazolone i.r.	↓ body weight loss	(196)
	
	0.005–0.0025% with PMA	PMA-induced inflammation in colon epithelial cell line Caco-2 PMA 300 ng/ml for 6 h	↓ secretion of IL-8	(196)

Grape seed PACs	400 mg/kg/day for 10 days	DSS in drinking water *ad libitum* From days 5–10	↓ ileal villus height ↓ mucosal thickness ↓ histological severity score in the distal ileum and in proximal colon	(197)

PACs-rich grape seed extract	100, 200, and 400 mg/kg/day for 7 days after first TNBS injection	Twice i.r. injection First 100 mg/kg TNBS, after 4 days 75 mg/kg	↓ colonic weight/length ↓ body weight loss ↓ microscopic and macroscopic damage score ↓ MPO activity ↓ lipid perocidation ↓ IL-1β levels ↑ IL-2 and IL-4 levels	(201)
	
	100, 200, and 400 mg/kg/day for 7 days after first TNBS injection	Twice i.r. injection First 80 mg/kg TNBS, after 16 days 30 mg/kg	↓ body weight loss ↓ microscopic and macroscopic damage score ↑ SOD and GSHpx activity ↑ GSH levels ↓ expression of TNF-α, p-IKKα/β, p-IκBα ↓ translocation NF-κB	(204)
	100, 200, and 400 mg/kg/day for 7 days after second TNBS injection			(203)
	
	200 mg/kg/day for 7 days after second TNBS injection	Twice i.r. injection First 80 mg/kg TNBS, after 16 days 30 mg/kg	↓ colonic weight/length ↓ body weight loss ↓ microscopic and macroscopic damage score ↓ MPO and iNOS activity ↓ lipid perocidation ↓ Nitric oxide levels ↑ SOD and GSHpx activity ↑ GSH levels	(202)

*DSS, dextran sulfate sodium; IEL, intraepithelial lymphocytes; iNOS, inducible nitric oxide synthase; GSH, glutathione; GSHpx, glutathione peroxidase; IL, interleukin; i.r. intrarectally; PMA, phorbol 12-myristate 13-acetate; SOD, superoxide dismutase; TNBS, 2,4,6-trinitrobenzene sulfonic acid; TNF-α, tumor necrosis factor alpha*.

The oral administration of apple (mainly containing B1, B2, and C1 PACs) or grape seed extract (with 75% PACs: 40% polymers, 14% dimers, 12% trimers, 8% tetramers) has been shown to prevent colonic damage in an experimental colitis model induced by dextran sodium sulfate or oxazolone in rodents (196–199). Accordingly, the mortality rate of the treated animals as well as their weight loss was lower, compared with those receiving the placebo. The grape extract also contributes to the normalization of the ileal villus morphology and mucosal thickness, reducing the histological severity score in the distal ileum and proximal colon (197). It has been postulated that one of the mechanisms implicated in these effects should be the modulation of the proportions of the TCR_γδ_/TCR_αβ_ intraepithelial lymphocytes. Indeed, these cells play a key role in the maintenance of mucosal homeostasis, contributing to the modulation of the activated release of pro-inflammatory cytokines by epithelial cells and, therefore to the prevention of inflammatory states in the mucosa (196, 200).

Grape seed extract (containing PACs >95%, dimeric >1.8%, oligomers >60%) also exerted a protective effect in recurrent colitis induced by the intracolonic injections of 2,4,6-trinitrobenzene sulfonic acid, reducing the colonic weight/length ratio and the microscopic and macroscopic damage scores (201–204). Such effect relied on the antiinflammatory and antioxidant properties of the extract, as it was shown to inhibit the NF-κB signaling pathway, reducing the expression levels of TNF-α, p-IKKα/β, p-IκBα, and the translocation of NF-κB to the nucleus of colonic epithelial cells (203, 204). As a consequence of these events, the animal treated with the extract exhibited a lower neutrophil infiltration in their colonic mucosa, a decrease of IL-1β concentration, lipid peroxidation, and colonic inducible nitric oxide synthase activity, while the concentrations of IL-2 and IL-4, the antioxidants enzymes activities, and the levels of glutathione increased. These effects were comparable to those obtained with sulfasalazine, the standard drug used for IBD treatment (201–204).

The antiinflammatory properties of polymeric PACs from *Pistacia vera* L. nuts and apple has been studied in cell models (Caco-2 and T84 cell lines) simulating some conditions of IBD through their activation by pro-inflammatory cytokines such as IL-1β or IFN-γ/IL-1β/TNF-α (205, 206). Apple and pistachio PACs was shown to prevent the cytokine-induced translocation of NF-κB to the nucleus and the subsequent secretion of pro-inflammatory cytokines by these epithelial cells. More specifically, B1 PAC inhibited in a dose-dependent manner the expression of pro-inflammatory genes and repressed NF-κB-, IP-10-, and IL-8-promoters and STAT1-dependent signal transduction (206). The PAC extract from pistachio also contributes to conserve the integrity of IL-1β-stimulated Caco-2 cell monolayers by attenuating the disruption of tight-junctions, preventing the drop in transepithelial electrical resistance and the alterations of the gut barrier function (205).

In a recent *in vitro* study, we investigated the protective effect of PAC-containing polyphenol extracts from apple, avocado, cranberry, or grape and PACs microbial metabolites on the deleterious effect induced by p-cresol in human colonic epithelial cells (HT-29 and Caco-2). In HT-29 cells, the cranberry and avocado extracts prevented the loss in cell viability (measured as lactate dehydrogenase leakage) and the diminution in ATP contents, while bacterial metabolites only prevented the loss in cell viability. In Caco-2 cells, all extracts and bacterial metabolites prevented the p-cresol-induced alterations of barrier function (measured as transepithelial electrical resistance and fluorescein-dextran transport). These results suggest that PAC-containing polyphenol extracts and PAC metabolites likely contribute to the protection of the colonic mucosa against the deleterious effects of p-cresol (207).

### Protective Effect of PACs on Colorectal Cancer

Colorectal cancer is strongly related to dietary habits and is one of the most common cancers worldwide (208). Many studies have assessed the impact of flavonoids including PACs on the risk of this cancer (11, 209–212). *In vitro* studies using human cells lines derived from colonic adenocarcinoma have reported an inhibitory effect of PACs on cell proliferation and growth (213–215), an increase of apoptosis associated to caspase 3 (213, 215, 216) or caspase 8 activation (217), an arrest of the cell cycle in G1 phase (215), or the suppression of the angiogenic factors vascular endothelial growth factor and angiopoietin 1 (in a model of tumor growth in a xenografted chick chorio-allantoic membrane) (218). In animals, grape seed PACs (0.1–1.0% of diet) inhibited by 72–88% the formation of aberrant colonic crypt foci induced by azoxymethane and by 20–56% the activity of ornithine decarboxylase, an enzyme involved in cell growth and differentiation and related to tumor promotion, in the distal colon (219). In another study using the same model, the administration PACs (0.002%) and PC B2 (0.05%) decreased aberrant crypt foci formation and cell proliferation in the colonic epithelium and increased apoptosis compared with control rats (220).

In humans, a case–control study carried out in Italy reveals a decreased risk of colorectal cancer in the subjects with higher intakes of PACs [odds ratio (OR) = 0.74], being this effect more effective for rectal than for colon cancer (221). Another case–control study in Scotland showed a reduction in the risk of colorectal cancer with the consumption of catechin (OR = 0.68 for the highest vs lowest quartile), epicatechin (OR = 0.74), and PCs (OR = 0.78) (222), while a study in Spain showed a decreased risk in the quartile of highest intake of PAC compared with that of lowest intake [OR = 0.58 CI_95%_ (0.35–0.96), *p* = 0.02]. In this study, PACs were mainly brought by fruits, wine, and legumes and similar to the Italian study, the protective effect of PACs was more pronounced for rectal than for colon cancer (223). In opposition with these studies, another Scottish case–control study and the prospective Iowa Women’s Health Study did not detect any association between PAC intake and colorectal cancer (224, 225) and Bobe et al., in 1,859 participants of the Polyp Prevention Trial, reported an association between PAC intake and the risk of colorectal adenoma recurrence in men (226).

In conclusion, although *in vitro, in vivo*, and epidemiologic studies suggest that PACs could act as a chemopreventive agent, further studies are necessary to confirm these results and to clearly establish the subjacent mechanisms implicated and the type and concentration of PACs involved in the protection, prior to use them for the prevention or management of colorectal cancer.

## Conclusion

During the postprandial period, high amounts of undigested dietary PACs are found in the gut lumen where they exert great number of activities beneficial for the health host. They contribute to the host defense against pathogens, the modulation of gastric emptying, the inhibition of emetic reflex, and the modulation of the composition of the IM in the colon, in a prebiotic-like manner. They also decrease the inflammatory and pro-oxidant processes occurring in the gastric and colonic mucosa, favoring ulcer healing and contributing to the GI mucosa integrity. In the small intestine, they interfere with the digestion and absorption of carbohydrates, proteins, lipids, and eventually LPS, and can modulate the secretion of GI hormones, the epithelial transport of water and electrolytes, and the GI transit. In the colon, PAC could act reducing the risk of colorectal cancer. Their degradation by the gut microbiota generates several metabolites with protective properties for the colonic epithelium and, when absorbed, for the extra-intestinal tissues. The numerous properties of PACs in the GI tract probably represent a large part of their overall effects on human health and contribute to explain the impact of the consumption of fruits and vegetables against the non-communicable chronic diseases. Due to these properties, they could eventually be used for the dietary management of several GI diseases, or as complementary treatment to attenuate adverse effects associated with the administration of certain drugs. However, further investigations are necessary to fully understand the mechanisms and effects of their use, as well as the doses and formulations necessary to generate the desired effects. Finally, since the beneficial effects of PACs are not only limited to humans, as shown by their positive effects in ruminant nutrition; accordingly, PACs could also be used to improve the productivity in livestock breeding and the healthy properties of animals products.

## Author Contributions

MC contributed to Sections “Introduction,” “Effect on GI Hormones Secretion and Gastric Emptying,” “Antiemetic Properties,” “Effects of PACs in the Digestion and Absorption of Nutrients,” “Effects of PACs in Diarrhea and GI Motility,” “The PACs in the Colon,” “Effect on the Intestinal Microbiota,” “Protective Effect of PACs on Colorectal Cancer,” and “Con-clusion.” XW contributed to Sections “Absorption of Flavan-3-ols and PACs,” “Intestinal Metabolism and Bioavailability of Flavan-3-ols and PACs,” and “Transformation of PACs by Gut Microbiota.” CC-P contributed to Sections “PACs in the Stomach,” “Protective Effect against *Helicobacter pylori (H. pylori)*,” “Protective Effect against Gastric Inflammation,” and “Protective Effect of PACs in Colonic Inflammation.” MG contributed to Sections “PACs in the Mouth,” “Effect on GI Hormones Secretion and Gastric Emptying,” “Antiemetic Properties,” “Absorption of Flavan-3-ols and PACs,” “Intestinal Metabolism and Bioavailability of Flavan-3-ols and PACs,” “Effects of PACs in Diarrhea and GI Motility,” “Protective Effect of PACs on Colorectal Cancer,” and “Conclusion.”

## Conflict of Interest Statement

The authors declare that the research was conducted in the absence of any commercial or financial relationships that could be construed as a potential conflict of interest.

## Abbreviations

PACs, proanthocyanidins; DP, degree of polymerization; GI, gastrointestinal; *Helicobater pylori, H. pylori*; VacA, vacuolating cytotoxin A; HMW, high molecular weight; LMW, low molecular weight; NSAIDs, non-steroidal antiinflammatory drugs; NOS, nitric oxide synthase; iNOS, inducible nitric oxide synthase; SH, sulfhydryl compounds; EGF, epidermal growth factor; PCNA, proliferating cell nuclear antigen; NO, nitric oxide; GLP-1, glucagon-like peptide-1; SGLT1, sodium–glucose cotransporter 1; GLUT, glucose transporter; TG, triglycerides; CS, cholesterol; FFAs, free fatty acids; IBAT, ileal bile acid transporter; AUC, area under the curve; NPC1L1, Niemann–Pick C1-like protein 1; LPS, lipopolysaccharide; IM, intestinal microbiota; SCFAs, short chain fatty acids; IBD, inflammatory bowel diseases; OR, odds ratio.
